# Digital interventions to moderate alcohol consumption in young people: a Cancer Prevention Europe overview of systematic reviews

**DOI:** 10.3389/fdgth.2023.1178407

**Published:** 2023-05-23

**Authors:** Kevin T. McDermott, Caro Noake, Robert Wolff, Carolina Espina, Jérôme Foucaud, Karen Steindorf, Joachim Schüz, Mangesh A. Thorat, Matty Weijenberg, Linda Bauld, Jos Kleijnen

**Affiliations:** ^1^ Kleijnen Systematic Reviews Ltd., York, United Kingdom; ^2^ Environment and Lifestyle Epidemiology Branch, International Agency for Research on Cancer/World Health Organisation (IARC/WHO), Lyon, France; ^3^ Institut National du Cancer (INCa), Boulogne-Billancourt, France; ^4^ Université Sorbonne Paris Nord, Laboratoire Éducations et Pratiques de Santé (UR 3412), France; ^5^ Division of Physical Activity, Prevention and Cancer, German Cancer Research Center (DKFZ) and National Center for Tumor Diseases (NCT) Heidelberg, Heidelberg, Germany; ^6^ Breast Services, Guy's Hospital, Guy’s and St Thomas’ NHS Foundation Trust, Great Maze Pond, London, United Kingdom; ^7^ Centre for Cancer Prevention, Wolfson Institute of Population Health, Queen Mary University of London, London, United Kingdom; ^8^ School of Cancer & Pharmaceutical Sciences, Faculty of Life Sciences & Medicine, King's College London, London, United Kingdom; ^9^ Department of Epidemiology, GROW School for Oncology and Reproduction, Maastricht University, Maastricht, Netherlands; ^10^ Usher Institute and SPECTRUM Consortium, University of Edinburgh, Edinburgh, United Kingdom

**Keywords:** digital health, cancer, systematic reviews, public health, evidence synthesis, alcohol consumption, evidence assessment

## Abstract

**Background:**

Strategies to reduce alcohol consumption would contribute to substantial health benefits in the population, including reducing cancer risk. The increasing accessibility and applicability of digital technologies make these powerful tools suitable to facilitate changes in behaviour in young people which could then translate into both immediate and long-term improvements to public health.

**Objective:**

We conducted a review of systematic reviews to assess the available evidence on digital interventions aimed at reducing alcohol consumption in sub-populations of young people [school-aged children, college/university students, young adults only (over 18 years) and both adolescent and young adults (<25 years)].

**Methods:**

Searches were conducted across relevant databases including KSR Evidence, Cochrane Database of Systematic Reviews (CDSR) and Database of Abstracts of Reviews of Effects (DARE). Records were independently screened by title and abstract and those that met inclusion criteria were obtained for full text screening by two reviewers. Risk of bias (RoB) was assessed with the ROBIS checklist. We employed a narrative analysis.

**Results:**

Twenty-seven systematic reviews were included that addressed relevant interventions in one or more of the sub-populations, but those reviews were mostly assessed as low quality. Definitions of “digital intervention” greatly varied across systematic reviews. Available evidence was limited both by sub-population and type of intervention. No reviews reported cancer incidence or influence on cancer related outcomes. In school-aged children eHealth multiple health behaviour change interventions delivered through a variety of digital methods were not effective in preventing or reducing alcohol consumption with no effect on the prevalence of alcohol use [Odds Ratio (OR) = 1.13, 95% CI: 0.95–1.36, review rated low RoB, minimal heterogeneity]. While in adolescents and/or young adults who were identified as risky drinkers, the use of computer or mobile device-based interventions resulted in reduced alcohol consumption when comparing the digital intervention with no/minimal intervention (−13.4 g/week, 95% CI: −19.3 to −7.6, review rated low RoB, moderate to substantial heterogeneity).In University/College students, a range of E-interventions reduced the number of drinks consumed per week compared to assessment only controls although the overall effect was small [standardised mean difference (SMD): −0.15, 95% CI: −0.21 to −0.09]. Web-based personalised feedback interventions demonstrated a small to medium effect on alcohol consumption (SMD: −0.19, 95% CI: −0.27 to −0.11) (review rated high RoB, minimal heterogeneity). In risky drinkers, stand-alone Computerized interventions reduced short (SMD: −0.17, 95% CI: −0.27 to −0.08) and long term (SMD: −0.17, 95% CI: −0.30 to −0.04) alcohol consumption compared to no intervention, while a small effect (SMD: −0.15, 95% CI: −0.25 to −0.06) in favour of computerised assessment and feedback vs. assessment only was observed. No short-term (SMD: −0.10, 95% CI: −0.30 to 0.11) or long-term effect (SMD: −0.11, 95% CI: −0.53 to 0.32) was demonstrated for computerised brief interventions when compared to counsellor based interventions (review rated low RoB, minimal to considerable heterogeneity). In young adults and adolescents, SMS-based interventions did not significantly reduce the quantity of drinks per occasion from baseline (SMD: 0.28, 95% CI: −0.02 to 0.58) or the average number of standard glasses per week (SMD: −0.05, 95% CI: −0.15 to 0.05) but increased the risk of binge drinking episodes (OR = 2.45, 95% CI: 1.32–4.53, review rated high RoB; minimal to substantial heterogeneity). For all results, interpretation has limitations in terms of risk of bias and heterogeneity.

**Conclusions:**

Limited evidence suggests some potential for digital interventions, particularly those with feedback, in reducing alcohol consumption in certain sub-populations of younger people. However, this effect is often small, inconsistent or diminishes when only methodologically robust evidence is considered. There is no systematic review evidence that digital interventions reduce cancer incidence through alcohol moderation in young people. To reduce alcohol consumption, a major cancer risk factor, further methodologically robust research is warranted to explore the full potential of digital interventions and to form the basis of evidence based public health initiatives.

## Introduction

Cancer is a leading cause of mortality in all European countries and the impact on individual health and wider society is significant. Studies have shown that nearly 40% of cancer cases are related to known modifiable risk factors, and therefore preventable (1). These known main risk factors include (but are not limited to) tobacco and excessive alcohol consumption, consequences of an unhealthy diet, being overweight and being sedentary with insufficient physical activity (2).

Alcohol consumption remains as one of the four leading causes of premature death, and the second leading cause of premature mortality in the World health Organisation (WHO) European region (3) (Supplementary File S4 for full list of abbreviations). It is well established that there exists a direct relationship between consumption of alcohol and the development of several cancers, such as those of the oral cavity, oropharynx, oesophagus, larynx and liver (4). Although the potential negative health effects e.g., increased risk of liver disease, cardiovascular disease, road accidents of alcohol are widely known, it is less well known that it is a risk factor for cancer, and that those who routinely indulge in heavy drinking are more at risk. Younger people in adolescence and during early adulthood are particularly vulnerable to the impact of alcohol consumption in general. Effective strategies to inform and educate younger people about the risks of alcohol consumption, may have considerable positive impact on both current and future health problems, including cancer incidence.

A recent WHO report stated that it is expected that most younger people tend to begin drinking alcohol between the ages of 12–16 (3) with drinking behaviours during adolescence associated with a multitude of physical, psychological, and social problems that can persist into later life. Drinking behaviours amongst university and college students has also been shown to be highly concerning, with risky drinking being common place (5) and heavy drinking being reported in university students in high income countries (6–8). Increased consumption of alcohol in younger people has been linked to increased alcohol consumption in later adulthood (9) which in turn has also has been linked to increased risk of cancer (10).

By promoting health-conscious behaviours, and increasing risk awareness around alcohol consumption, young people will be more informed and healthy lifestyle choices can be made. This could lead to substantial public health improvement with reductions in health and social problems associated with alcohol consumption, both now and in the future The increasing popularity, accessibility, and multi-functional use of digital technologies (computer, mobile phone, tablet etc.) make these potential tools to help facilitate communication, education, and risk awareness to elicit protective changes in behaviour, especially among children, adolescents and young adults, who are generally more familiar with new technologies throughout their formative years. young people aged between 10 and 24 years who are approaching adolescence and early adulthood may therefore be particularly suitable as recipients for such digitally delivered interventions. Mobile Health (mHealth) initiatives for instance, have rapidly expanded and are being utilised to deliver public health interventions, especially in the younger population who conduct many of their daily activities using smartphones and have been termed the “phono-sapiens” (11, 12). The flexibility of such mHealth platforms provide opportunities for public health specialists to target a large number of people and also monitor people's behaviour in “real-time” (13), and further emphasise the potential of digital technology in healthcare delivery It has previously been suggested that by addressing interventions for those who demonstrate the riskiest drinking behaviours, the greatest outcomes at the population level can be realised (14) and we are conscious that such digital technologies may have considerable impact in helping to moderate alcohol consumption in younger people.

Considering the negative public health and social impact that alcohol has, including increased risk of alcohol related cancers; alongside the prevalence of hazardous and harmful drinking behaviours in younger populations, we were interested in examining the impact and accessibility of emerging digital technology to moderate drinking behaviours in those younger populations. We reviewed the available systematic review literature with the objectives to ascertain (1) are digital interventions aimed at young people effective in addressing alcohol consumption? and (2) What is the quality and strength of the systematic review evidence?

## Methods

**Figure 1:**
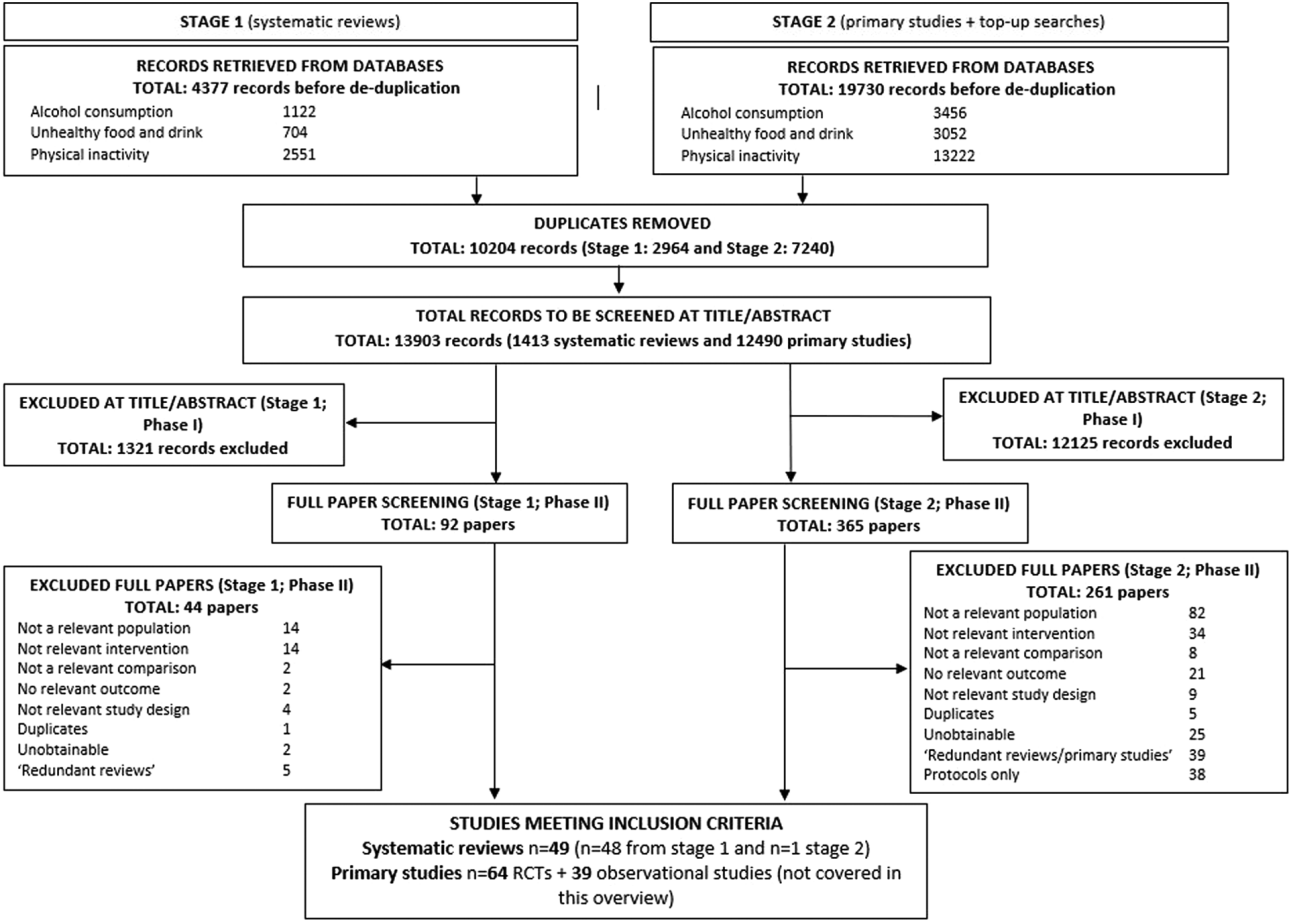
PRISMA flow chart. Literature searches of the wider project (including the topics: unhealthy food and drink, alcohol consumption, and physical activity and inactivity). Alcohol relevant systematic reviews discussed in this article represent 27 included systematic reviews.

This paper addressing the systematic review evidence for digital interventions and impact on alcohol consumption, has emerged from a wider project investigating the impact of digital technologies on a variety of behavioural risk factors. For this reason, search strategies (Supplementary File S1), excluded studies (Supplementary File S2) and specific numerical data in the PRISMA flow chart (Figure 1) are broader than the topic of alcohol alone. Other areas of interest included unhealthy food and drinks, and physical activity and inactivity. Due to the large overlap between these topics and to ensure completeness all search results were imported into a single Endnote library and screened for all areas of interest.

### Eligibility criteria

Reviews were selected for inclusion based on the following criteria:

Children, adolescents and young adults aged 10–24 years, including mean age within this range or a subgroup within this range. The age range of 10–24 years was selected as this represented the age range of full-time school and university level education, people in this age range were more routinely exposed to digital technology, and where behavioural and lifestyle patterns were being formed. We have considered children to be aged 10–16 years, adolescents to be 16–18 years, and young adults to be aged 18–24 years. However, we emphasise that these are not absolute definitions, and some studies may include participants that can overlap into more than one category.

• Population: Children, adolescents and young adults of school and university/college age (aged 10–24 years). Other combinations of age subgroups were also included, such as: school-aged children [includes adolescents; ≤18 years]; college/university students; young adults [≥19 years]; both adolescents and young adults [any age range <25]
• Intervention: Digital interventions addressing alcohol consumption. The definition of digital interventions followed that of the WHO which includes targeted client communication, untargeted client communication, client to client communication, personal health tracking and on-demand information services to clients (15). All interventions delivered by a healthcare or other professional or peer as well as those intended to be self-guided were included. A digital intervention was generally understood to be delivered primarily through programmable computer or mobile device (laptop, mobile phone, tablet, or smart watch).It should be noted that a device (computer, mobile phone, tablet etc.), could be used to receive intervention via internet (email, apps, website login) or phone network connectivity (SMS, MMS) and these distinctions should be considered when reviewing the evidence presented here, i.e. digital or internet may be synonymous and interchangeable with mobile phone or computer. It is important to note that some interventions could fit into more than one category and the final classification and grouping in this article was based on reviewers' opinions and discussions.
• Comparators: Any comparators were eligible. This included studies where the control group received no intervention, is on a waiting list or received an active intervention (digital or non-digital such as printed or face-to-face).
• Outcomes: Self-reported or objective measures related to reduction of alcohol. Reduction in cancer incidence because of the interventions (if available) was eligible. Relevant outcomes were those relating to quantity, frequency, and intensity of alcohol consumption. Adverse events (unintended consequences) relating to the interventions were also of interest.
• Systematic reviews were eligible. This included any study labelled by the study authors as a systematic review irrespective of quality.

### Literature search and screening

Each area of interest in the wider project, including alcohol consumption was addressed with separate strategies, which were structured using search terms for general and question-specific digital interventions. The overall search strategy for the broader project was conducted in two stages. During stage 1, a rapid appraisal to identify existing systematic reviews and health technology assessments (HTAs) was conducted.

The following databases and organisational websites were searched in April 2021 for relevant reviews, from database inception to present (see Supplementary File S1):

• KSR Evidence (www.ksrevidence.com);
• Cochrane Database of Systematic Reviews (CDSR) (Wiley).
• Database of Abstracts of Reviews of Effects (DARE) (CRD).
• Health Technology Assessment Database (HTA)(CRD).
• Epistemonikos (https://www.epistemonikos.org/).

Additionally manual searching of the following resources was conducted by reviewers to identify any relevant publications.

• World Cancer Research Fund (WCRF) (https://www.wcrf-uk.org/).
• International Agency for Research on Cancer (IARC) (https://www.iarc.fr/).
• World Health Organization (WHO) (https://www.who.int/health-topics/cancer).

Once the main relevant systematic reviews and HTA evidence were identified for each research question, a series of more focused rapid review searches were carried out (stage 2). Appropriate date limits were defined in relation to each topic's systematic reviews evidence base (2016 for alcohol consumption and 2015 for unhealthy food and drink and physical inactivity). Where a relevant systematic review had a latest search date before 2016, it was considered as possibly ‘out-of-date’ as it would be likely to detail technology that has been surpassed by newer developments, or to contain superseded primary research. The following databases were searched for relevant studies:

• MEDLINE (Ovid).
• MEDLINE In-Process Citations, Daily Update & Epub Ahead of Print (Ovid).
• Embase (Ovid).

Search strategies were developed specifically for each database and the keywords adapted according to the configuration of each database (see Supplementary File S1). Due to the broad nature of the wider topic the review team recognised that the free text terms included in the strategies were not exhaustive, but the combination of the use of subject headings where available and the checking of reference lists in included studies was used to reduce the loss of recall. Searches were not limited by language or publication status (unpublished, published, in press, and in progress).

Titles and abstracts identified through electronic database and web searching were independently screened by two reviewers. Subsequently, full texts were independently examined in detail by two reviewers to determine whether they met the criteria for inclusion in the wider research project (see Supplementary File S2 for details of studies excluded at this stage). Any discrepancies between reviewers were resolved through discussion or the participation of a 3rd reviewer. At this phase, articles were categorised by the specific research question they addressed, in this case by alcohol consumption. The study selection process is detailed in accordance with the Preferred Reporting Items for Systematic Reviews and Meta-Analyses (PRISMA) statement (16).

### Data extraction

Data extraction was also performed by teams of two reviewers. One reviewer identified and extracted the data, and a second reviewer checked the extracted data against the original review. Any discrepancies were resolved through discussion with a third reviewer.

Extracted data comprised of basic information [author, year, years range and number of relevant primary studies, review type (alcohol), intervention type, search end date, type of included study designs, best data available], information on population, intervention, comparator, and outcomes (PICO) and the overall conclusions.

### Rob assessment

The RoB was assessed using the Risk of Bias Assessment Tool for Systematic Reviews (ROBIS) (17). Two reviewers independently assessed study quality and any discrepancies were resolved through discussion and consensus or the intervention of a third reviewer.

### Statistical analyses

A narrative summary of the included systematic reviews is presented with a summary of the main study characteristics tabulated. No additional quantitative data synthesis was performed.

Emphasis was put on recent reviews, reviews of higher quality based on ROBIS scores and reviews where meta-analysis was conducted. Where reviews carried out a relevant meta-analysis, the pooled results were included. Conclusions from qualitative and/or older reviews were briefly summarised in narrative. Given the rapidly developing technology that exists, reviews were considered as possibly out-of-date if they had a latest search date before 2016. However, where other evidence was limited, these older reviews were included and variously introduced.

The reviews were categorised based on (1) the type of population as described in the paper or based on age provided in the paper (school-aged children [includes adolescents; ≤18 years]; college/university students; young adults [≥19 years]; both adolescents and young adults [any age range <25]) and (2) type of intervention [mobile phone; computer only; internet only; games; digital (any); other]. Where a review reported on a range of different digital interventions we defined these within the category ‘any digital'.

## Results

### Characteristics of included reviews

The stage 1 systematic literature search for systematic reviews retrieved a total of 4,377 records, with 1,122 being relevant to alcohol. The stage 2 systematic search identified 19,730 records, with 3,456 being relevant to alcohol. After de-duplication and screening 49 systematic reviews were identified for the broader project area. Of these, 25 systematic reviews (13, 18–41) met the alcohol relevant inclusion criteria. An additional two relevant systematic reviews (42, 43) were identified from further searching resulting in a total of 27 systematic reviews (Table 1), included in our review of reviews.

**Table 1:** Characteristics of included systematic review.

Review	Type of synthesis	Stated aims	Inclusion/exclusion criteria	No of studies^a^	Relevant main outcomes	RoB	Searches conducted up to^b^	Review overall conclusions	Relevant points (“Take home” summary)
**School-aged children**									
**Digital Intervention (Any)**									
Champion et al. (18)	Qualitative	To address these gaps in the literature by reviewing the evidence on, and establishing whether school-based prevention programs facilitated by the computer, or the Internet have the potential to reduce and prevent the use of alcohol and other drugs in adolescents	*Design*: RCTs *Participants*: Adolescents (school- aged, most 13–15) *Intervention:* Internet-based and computer-based prevention program for alcohol (or other drugs) delivered at school. *Comparator:* Not stated	6	Alcohol consumption (quantity, intensity)	High	March 2012	The implementation advantages and high fidelity associated with new technology, suggest that programs facilitated by computers and the Internet offer a promising delivery method for school-based prevention.	High RoB, no meta-analysis was conducted, Search date of March 2012 suggests older research utilising older technologies. Largely superseded by champion 2019 and not prioritised for discussion.
Champion et al. (43)	Meta-analysis	To systematically review eHealth school-based interventions targeting alcohol use and other lifestyle factors and identify intervention characteristics associated with effectiveness	*Design*: RCTs (including randomisation at the school grade or class level) *Participants*: students aged 11–18 years. *Intervention:* School based prevention programme delivered via eHealth methods (e.g., the internet, computers, tablets, mobile technology, or tele-health, *Comparator*: No intervention, education as usual, or an alternate evidence-based intervention not delivered via eHealth (eg, face to face).	25	Alcohol consumption (prevalence)	Low	14 March 2019	eHealth school-based interventions addressing multiple lifestyle risk behaviours. eHealth school-based multiple health behaviour change interventions were not effective in preventing or reducing alcohol use.	Well conducted review with Low RoB, utilised RCT evidence, searches up to March 2019 suggest relatively recent research utilising up to date technologies, Overall findings suggest that no effect observed on alcohol outcomes. Based on 6 studies reporting alcohol use outcomes. 2 studies, with 4 intervention groups, were combined in the meta-analysis showing interventions had no effect on the prevalence of alcohol use (OR = 1.13, 95% CI: 0.95–1.36). Limited relevant research identified.
**College/University students**									
**Computer**									
Elliot et al. (19)	Qualitative	To examine the results of randomized controlled trials of e-interventions designed to reduce college drinking.	*Design*: RCTs *Participants*: College students *Intervention:* Computer based intervention *Comparator*: Not stated	19	Alcohol consumption (quantity, frequency, intensity), blood alcohol concentration, alcohol problems	High	August 2007	Overall, findings provide some support for such programs, especially in comparison with assessment-only control conditions. In addition, possible moderators (e.g., baseline drinking patterns) and mediators (e.g. corrected drinking norms) have emerged.	Review included searches up to August 2007 therefore considered to likely include outdated technology and research, review had high RoB, no meta-analysis was conducted. Not prioritised for discussion.
**Digital Intervention (Any)**									
Carey et al. (20)	Meta-analysis	To evaluates the efficacy and moderators of computer-delivered interventions (CDIs) to reduce alcohol use among college students.	*Design*: Not stated, *Participants*: Undergraduate students, *Intervention:* Alcohol-related intervention delivered via computer or electronic device, *Comparator:* Not stated	33	Alcohol consumption (quantity, frequency, intensity), alcohol-related problems	High	Not stated	CDIs reduce the quantity and frequency of drinking among college students. CDIs are generally equivalent to alternative alcohol-related comparison interventions.	Review did not confirm search dates and therefore considered to likely include outdated technology and research, review had high RoB and was not prioritised for discussion.
Dick et al. (24)	Qualitative	To systematically identify and critically appraise studies examining the effectiveness of digital interventions for, illicit substance misuse and harm reduction	*Design*: Not stated *Participants*: College students *Intervention:* Web-based or mobile digital intervention with the aim of reducing harm from substance misuse, excluding alcohol-specific interventions *Comparison*: Not stated	8	Alcohol consumption (quantity)	High	April 2018	The results support the need for further research, particularly given the success of digital interventions for alcohol and tobacco harm reduction.	Overall conclusions did not relate to alcohol, and they did not present a meta-analysis. Only 4 studies were relevant to alcohol outcomes. No study designs were clarified and there was high RoB meaning it was not prioritised for discussion.
Prosser et al. (25)	Meta-analysis	To evaluate the effectiveness and moderators of E-Interventions vs. assessment only controls in the reduction of alcoholic drinks per week in university students	*Design*: RCTs *Participants*: College students *Intervention:* E-intervention, in that the intervention was delivered via a technological device *Comparator*: Not stated	21	Alcohol consumption (quantity)	High	June 2017	E-Interventions show a small, significant effect at reducing mean alcoholic drinks per week. Personalised feedback E-Interventions showed the strongest effect	E-interventions reduced the number of drinks per week compared to assessment only controls, (SMD: −0.15, 95% CI: −0.21 to −0.09). Web-based personalised feedback interventions demonstrated a small to medium effect on reducing drinks (SMD: −0.19, 95% CI: −0.27 to −0.11). For the other interventions, there was no effect (SMD: −0.07, 95% CI: −0.14 to 0.00). Searches dating only up to June 2017, so the most recent literature on has not been evaluated. High RoB.
**Internet**									
Bhochhibhoya et al. (21)	Qualitative	To analyse available evidence by utilising established systematic review techniques to provide systematic appraisal of the Internet-based interventions aimed at reducing binge drinking among the college population	*Design*: Not stated *Participants*: College students *Intervention:* Internet-based interventions aimed at reducing binge drinking among the college population *Comparator:* Not stated	14	Alcohol consumption (quantity, frequency)	High	2014	This review supports using the Internet as a brief intervention approach that can effectively support efforts to reduce binge drinking among college students.	Encouraging results but high RoB, lack of clarity on included studies, and lack of meta-analysis, searches were up to 2014 and so included technology and research may be outdated.
Leeman et al. (22)	Qualitative	To address the efficacy of very brief web-based alcohol interventions	*Design*: RCTs *Participants*: College students *Intervention:* Very-brief, web-based alcohol reduction interventions *Comparator*: Not stated	15	Alcohol consumption (quantity, frequency, intensity), alcohol-related problems	High	September 2014	This review yielded some evidence supporting very-brief, web-based interventions in reducing alcohol use but not related problems in college students. Very-brief, web-based interventions are worth pursuing given their convenience, privacy, and potential public health benefit.	Considerable variation between included trials across study design, outcomes and interventions mean that reliability in overall conclusions cannot be made. High RoB and no meta-analysis conducted. searches were up to 2014 and so included technology and research may be outdated.
Bedendo et al. (23)	Qualitative	To identify the main modalities of interventions via the Internet to limit the use of alcohol in university students and to describe the effects of these interventions on consumption and consequences of alcohol use.	*Design*: RCTs *Participants*: College students *Intervention:* Internet-based interventions. Two main intervention modalities were identified: personalised normative feedback and multicomponent interventions. *Comparator:* Not pre-specified	36	Alcohol consumption and consequences of alcohol consumption	High	February 2016	Personalised normative feedback and the AlcoholEdu website, the most frequently evaluated interventions among the selected studies, were effective in reducing alcohol use in university students.	Most up to date review describing internet based interventions in college students, had high RoB and no meta-analysis was conducted, searches were up to early 2016 and so included technology and research may be outdated.
**Young adults only**									
**Computer**									
Khadjesari et al. (26)	Meta-analysis	To determine the effects of computer-based interventions aimed at reducing alcohol consumption in adult, populations	*Design*: RCTs *Participants*: Adults (aged 18 years and older) *Intervention:* Stand-alone (non-guided) computer-based interventions *Comparator:* Minimally active (e.g., assessment-only, usual care, generic non-tailored information, or educational materials) or an active comparator group (e.g., brief intervention).	18	Alcohol consumption (quantity, frequency)	Low	December 2008	Computer-based interventions may reduce alcohol consumption compared with assessment-only; the conclusion remains tentative because of methodological weaknesses in the studies.	Meta-analyses suggested that computer-based interventions were more effective than minimally active comparator groups (e.g., assessment-only) at reducing grams of alcohol consumed per week in student (MD: −19.42, 95% CI: −29.83 to −9) and non-student populations (MD: −114.94, 95% CI: −198.6 to −31.29). This review was reliable but only covered studies up to December 2008 and therefore out-of-date technology and research is likely a concern.
**Digital intervention (Any)**									
O’Rourke et al. (27)	Qualitative	To review the efficacy of electronic based communication interventions for alcohol misuse amongst hazardous young	*Design*: RCTs observational studies *Participants:* Young adults (18 to 25 years) screened as being hazardous drinkers *Intervention*: Behavioural interventions delivered via electronic communication methods: (1) Web-based; (2) email; (3) text messages SMS and MMS and; (4) Social Network Sites *Comparator:* Treatment as usual, placebo, no intervention	13	Alcohol consumption (quantity, frequency, intensity), AUDIT	High	January 2016	Usage of text messaging and Social Network Sites (SNS) increased accessibility and ease of engaging in an intervention that is appealing and acceptable for young adults.	High RoB and no meta-analysis, differing intervention styles included, searches up to Jan 2016 suggest that more up to date research and technology may be missed.
**Both adolescents and young adults**									
**Computer**									
Rooke et al. (30)	Effect estimates	To quantify the overall effectiveness of computer-delivered interventions for alcohol and tobacco use	*Design*: RCTs *Participants*: Any *Intervention*: Computer or internet-based interventions *Comparator:* Not computer or internet-based interventions	20	Abstinence, change from preintervention to follow-up and post-intervention use	High	January 2009	Findings of the meta-analysis suggest that minimal contact computer-delivered treatments that can be accessed via the internet may represent a cost-effective means of treating uncomplicated substance use and related problems.	Computer-based interventions were effective at reducing alcohol but effects as reported by standardised differences were small (*d* = 0.22, 95% CI: 0.14–0.29). Final searches were conducted in January 2009 so up to date technology and research will likely be missed.
**Digital intervention (Any)**									
Kaner et al. (28)	Meta-analysis	To assess the effectiveness and cost effectiveness of digital interventions for reducing hazardous and harmful alcohol consumption, alcohol-related problems, or both, in people living in the community	*Design*: RCTs *Participants*: people living in the community whose alcohol consumption had been screened as hazardous or harmful (WHO 1992) and who were directed toward any digital intervention *Intervention*: digital, defined as being delivered primarily through a programmable computer or mobile device (laptop, phone or tablet), and were responsive to user input to generate personalised content which aimed to change the participants’ alcohol-related behaviours. *Comparator*: objectives 1 and 3 the control condition was no intervention (screening or screening and assessment only), printed or onscreen health or alcohol-related information, or in a health setting the care the patient would have received anyway for their presenting complaint. objective 2, the control condition was a face-to-face brief intervention to reduce alcohol consumption or harm.	26	Alcohol consumption (quantity, frequency, intensity), other measures of consumption, indices of alcohol harm or social problems to drinkers or affected others, cost-effectiveness, adverse events	Low	March 2017	There is moderate-quality evidence that digital interventions may lower alcohol consumption in hazardous drinkers. Substantial heterogeneity and risk of performance and publication bias may mean the reduction was lower. Low-quality evidence suggested there may be little or no difference between digital and face-to-face interventions., Behaviour change techniques were associated with effectiveness to reduce alcohol consumption.	High quality reliable Cochrane review, screened risky drinkers and clear definition of digital interventions although this included several different types of device, all were interactive with interventions being responsive to feedback. Focus of the review was not restricted to adolescents and young adults, so relevant results are limited. the difference between the digital intervention and no or minimal intervention arms in the quantity of alcohol consumed was smaller in magnitude than in the main analysis (−13.4 g/week, 95% CI: −19.3 to −7.6). Furthermore, this value differed significantly from the corresponding value in adults (aged >18 years) (−56.1 g/week, 95% CI: −82.1 to −30.0). Searches up to 2017 suggest that recent research or technology may be missed.
Dedert et al. (31)	Meta-analysis	To characterize treatment intensity and systematically review the evidence for efficacy of e-interventions, relative to controls, for reducing alcohol consumption and alcohol-related impairment in adults and college students	*Design*: RCTs *Participants*: Patients with alcohol misuse or an alcohol use disorder *Intervention:* E-interventions could be delivered by CD-ROM, online, mobile applications, or interactive voice response *Comparator:* Inactive or active controls	13	Alcohol consumption (quantity, frequency), alcohol problems, met limit guidelines	High	25 Mar 2015	Evidence suggests that low-intensity e-interventions can produce small reductions in alcohol consumption at 6 months, but there is little evidence for longer-term, clinically significant effects. Future e-interventions could provide more intensive treatment and possibly human support.	Meta-analysis relating to adolescents and young adults was in college students where e-interventions were associated with a small reduction in alcohol consumption at six-month follow-up (MD: −11.7 grams per week, 95% CI: −19.3 to −4.1). In five trials that used 12-month follow-up assessments analyses revealed no reduction in alcohol consumption (MD: −4.7 grams per week, 95% CI: −24.5 to 15.1). Interventions were largely varied and review had high RoB. Search dates of 2015 mean that up to date technology and research may be missed out.
Shingleton et al. (42)	Qualitative	To describe and evaluate the methods and efficacy of technology-delivered motivational interviewing interventions (TAMIs), discuss the challenges and opportunities of TAMIs, and provide a framework for future research	*Design*: Not stated *Participants*: Not reported *Intervention*: Technology-delivered motivational interviewing interventions *Comparator*: Not stated	5	Alcohol consumption (quantity, frequency), blood alcohol concentration, drunk driving (frequency)	High	27 February 2015	Researchers have used a range of technologies to deliver TAMIs suggesting feasibility of these methods. However, there are limited data regarding their efficacy, and strategies to deliver relational components remain a challenge. Future research should better characterize the components of TAMIs, empirically test the efficacy of TAMIs with randomized controlled trials and incorporate fidelity measures.	Review had a high RoB with minimal focus on alcohol consumption in adolescents and young people from included studies. Synthesis was qualitative and likely out of date given the February 2015 final search date.
Hutton et al. (13)	Qualitative	To examine current evidence on the effectiveness of mHealth technology use in positively influencing alcohol-related behaviours of young people without known alcohol addiction	*Design*: Not stated *Participants*: Adolescents and young adults (12–26 years) without alcohol dependency or a pre-existing condition related to alcohol *Intervention:* An mHealth intervention delivered via website or mobile technology (including text messages, Apps on smartphone devices, iPad and internet delivered treatment). *Comparator:* Not stated	17	Alcohol consumption (quantity, frequency)	High	January 2017	Use of mHealth, particularly text messaging was found to be an acceptable, affordable, and effective way to deliver messages about reducing alcohol consumption to young people. Further research using adequately powered sample sizes in varied settings, with adequate periods of intervention and follow-up, underpinned by theoretical perspectives incorporating behaviour change in young people's use of alcohol, is needed.	High RoB and no meta-analysis but most up to date in this sub-group. mhealth interventions were wide ranging and included studies varied in design, participant characteristics, settings, length and outcome measures. Reduction in alcohol consumption was reported in some studies but methodological limitations mean reliable and consistent conclusions cannot be made.
Haug et al. (35)	Qualitative	To review, the published literature on Internet and mobile, phone interventions to decrease alcohol consumption and for smoking cessation in adolescents	*Design*: RCT, quasi-RCTs and pre-post studies *Participants*: Adolescents and young adults from 12 to 25 formed the majority of participants *Intervention:* Internet-based and mobile phone interventions *Comparator:* Not stated	12	Alcohol abstinence or reduction	High	August 2009	Suggestions for the implementation of certain intervention approaches in Germany could not be derived from the existing studies. In the German context research was recommended to test the efficacy of web-based social norms interventions to decrease alcohol consumption in student and non-student samples.	Out of date with final searches in August 2009 meaning recent research and technology will be missed out. Review had high RoB and was therefore not prioritised.
Tebb et al. (36)	Qualitative	To conduct a literature review of computer-based interventions (CBIs) designed to address alcohol use among adolescents and young adults (aged 12–21 years) and examine the extent to which CBIs use theories of behaviour change in their development and evaluations.	*Design*: Not stated *Participants*: Adolescents and young adults (12–21 years) *Intervention:* Computer-based interventions aimed at preventing or reducing alcohol delivered via computer, tablet, or smartphone. *Comparator:* Not stated	Unclear	Alcohol consumption (quantity, frequency, intensity), blood alcohol concentration	High	December 2014	Given the importance of theory in guiding interventions, greater emphasis on the selection and application of theory is needed. The classification system used in this review offers a guiding framework for reporting how theory based principles can be applied to computer based interventions	Review had a high RoB with several methodological limitations and no meta-analysis was conducted. Focus was on how computer-based interventions integrate theories of behaviour change to address alcohol use among adolescents and young adults. The need for greater emphasis on the selection and application of theory in computer-based interventions was identified. Review was not prioritised for discussion.
Ohinmaa et al. (39)	Qualitative	To do a systematic review of telehealth studies in addiction/substance abuse and study the effectiveness and/or cost-effectiveness of the different telehealth applications in different addiction problems.	*Design*: Comparative studies, *Participants*: Any, *Intervention*: Internet-based, computer-based, text messages, *Comparator*: No treatment or non-telehealth treatment	Unclear	Alcohol consumption (quantity, intensity), problems	High	May 2009	The results of this review indicate that the Internet, computer, and telephone applications are at least as effective as conventional services, especially when we review more recently published applications that utilize personalized, interactive modular settings.	Focus was on telehealth for a range of substance abuse and addictive behaviours. Final searches were 2009 and so outdated with a high RoB. The review concluded that there are promising studies in internet applications for alcohol addiction when more developed interactive programs are used in motivated high risk/problem drinking populations. Review was not prioritised for discussion.
Smedslund et al. (40)	Meta-analysis	To assess the effectiveness of early, computerized brief interventions on alcohol and cannabis on high-risk users	*Design:* RCTs and quasi RCTs *Participants*: Young people (15–25 years) that were high/risky consumers of alcohol *Intervention:* Computerized brief intervention used as a stand-alone treatment *Comparator:* No intervention, waiting list control or an alternative brief intervention (computerized or non-computerized)	52	Alcohol consumption (quantity, frequency, intensity)	Low	April 2016	The interventions significantly reduce alcohol consumption in the short-term compared to no intervention, but the effect size is small, and there is no significant effect in the long-term. There are also shortcomings in the quality of the evidence. Interventions which provide an assessment of alcohol use with feedback may have a larger effect that those which do not, but again, the evidence is limited. There was no evidence of adverse effects.	Review was of low RoB and although interventions well defined, included a range of different modes. Results indicate Computerized interventions reduced short (SMD: −0.17, 95% CI: −0.27 to −0.08,) and long term (SMD: −0.17, 95% CI: −0.30 to −0.04) alcohol consumption compared to no intervention, while a small effect (SMD: −0.15, 95% CI: −0.25 to −0.06) in favour of computerised assessment and feedback vs. assessment only was observed. No short term (SMD: −0.10, 95% CI: −0.30 to 0.11) or long term effect (SMD: −0.11, 95% CI: −0.53 to 0.32) was demonstrated for computerised brief interventions when compared to counsellor based interventions.
Calverly et al. (41)	Qualitative	To assess effects of dietary advice given by a dietician compared with another health professional, or use of self-help resources, in reducing blood cholesterol in adults	*Design*: Not stated *Participants*: Adolescents and young adults (15–24 years) *Intervention*: Alcohol education programs, including through digital interventions *Comparator*: Not stated	37	Alcohol consumption (quantity, frequency, intensity), blood alcohol concentration,	High	June 2020	Findings indicated some education programs have capacity to positively change alcohol-related behaviour; however, outcome consistency varied even in high-quality programs. Alcohol education programs should be designed alongside health education/promotion models and best-practice recommendations, to improve the likelihood of desirable behaviour related outcomes.	Up to date, with a qualitative synthesis, but high RoB. The review assessed the quality of the interventions provided using ten criteria and concluded that some education programmes have the capacity to positively change alcohol-related behaviour; however, outcome consistency varied even in high-quality programmes.
**Internet**									
Bewick et al. (29)	Qualitative	To review the published literature on the effectiveness of web-based interventions designed to decrease consumption of alcohol and/ or prevent alcohol abuse	*Design*: Not stated *Participants*: Any *Intervention*: Web-based interventions for alcohol consumption *Comparator:* Not stated	5	Alcohol consumption (quantity, frequency, intensity), AUDIT	Low	May 2006	The current review provides inconsistent evidence on the effectiveness of electronic screening and brief intervention for alcohol use (in any/all populations). Process research suggests that web-based interventions are generally well received. However, further controlled trials are needed to fully investigate their efficacy and impact.	Review had a low RoB but was out of date with last searches being in May 2006 meaning up to date research and technology would be missed. They report that web-based personalized feedback alone compared to web-based feedback combined with additional self-help material sees the results favoured the combined intervention. Where web-based newsletters with no personalized component were compared to traditional print newsletters the results suggest that traditional print modes of delivery are more effective. However, when a web-based text education website without personalized feedback was compared to a personalized interactive website the results did not favour either intervention
Tait and Christensen (34)	Qualitative	To conduct a systematic review of randomised trials of web-based interventions for problematic substance use by adolescents and young adults.	*Design*: RCTs *Participants*: Adolescents and young adults (≤25 years) *Intervention:* Internet-based interventions *Comparator:* At least no treatment control	14	Alcohol consumption (quantity, frequency)	High	February 2009	Based on findings largely from tertiary students, web interventions targeting alcohol-related problems have an effect about equivalent to brief in-person interventions, but with the advantage that they can be delivered to a far larger proportion of the target population. Web-based interventions to prevent the development of alcohol-related problems in those who do not currently drink appear to have minimal impact.	No meta-analysis conducted and out of date with final searches being conducted early 2009 meaning up to date research and technology missed out. Review had high RoB.
Giroux et al. (37)	Qualitative	To summarize current knowledge regarding psychological interventions provided entirely online (via computers or mobile applications) for at risk or problem gamblers or users (alcohol, illegal drugs) and that were assessed for efficacy.	*Design*: Not stated *Participants*: Not stated *Intervention:* Online interventions targeting the reduction of behaviours or symptoms related to alcohol *Comparator*: Not stated	3	Alcohol consumption (quantity, frequency)	High	June 2015	Online interventions seem promising and appear to meet the needs of participants who are in the workforce and seeking help for the first time. Long-term efficacy studies should nonetheless be conducted.	Review only included three studies in the age group of interest for alcohol and did not have specific conclusions for this age group. Review was of high RoB and final searches in June 2015 mean recent technology and research may have been missed.
White et al. (38)	Qualitative	To review the efficacy of online interventions for alcohol misuse	*Design*: RCTs *Participants*: Not stated *Intervention*: Online interventions for alcohol misuse *Comparator*: Not stated	17	Alcohol consumption (quantity, frequency), blood alcohol concentration	High	December 2009	The available evidence suggests that users can benefit from online alcohol interventions and that this approach could be particularly useful for groups less likely to access traditional alcohol-related services. However, caution should be exercised given the limited number of studies allowing extraction of effect sizes, the heterogeneity of outcome measures and follow-up periods, and the large proportion of student-based studies. More extensive RCTs in community samples are required.	Out of date with high RoB and was not prioritised for discussion.
**Mobile telephone**									
Bastola et al. (32)	Meta-analysis	The main goal of this study was to analyse the effectiveness of mobile phone-based text messages as a preventive intervention for youth and younger adult populations’ problem drinking	*Design*: RCTs *Participants*: College students and young adults (<39 years) *Intervention:* Text message reminders for alcohol-related behavioural interventions. *Comparator:* Not stated	5	Alcohol consumption (quantity, frequency)	High	2018	The meta-analysis suggests that text message-based interventions might not be effective in decreasing alcohol intake in the younger populations.	Focused on college students and university students but defined young adults as under 39 years of age. Mobile phone text messages were less effective than controls at preventing binge drinking (OR = 2.45, 95% CI: 1.32–4.53) and were no different for mean reductions in drink per occasion (SMD: 0.28, 95% CI: −0.02 to 0.58) and average standard glasses per week (SMD: −0.05, 95% CI: −0.15 to 0.05). Longer-term effects were similar, favouring controls for reduction of binge drinking (OR = 7.24, 95% CI: 2.71–19.31). Review had a high ROB and although included interventions were all based on mobile phones text messages, size and frequency varied.
Staiger et al. (33)	Effect estimates	To conduct a systematic literature review of trials evaluating mobile app interventions for problematic tobacco, alcohol, and illicit drug use.	*Design*: Controlled trials *Participants*: Not stated *Intervention*: Mobile device apps (not web-based or SMS in isolation) *Comparator:* Any	20	Alcohol consumption (quantity, frequency)	High	01 February 2019	Although most app interventions were associated with reductions in problematic substance use, less than one-third were significantly better than the comparison conditions at post treatment. Moreover, 3 out of 6 apps included feedback and 2 had high- risk of bias, 1 some risk, and 3 low- risk. All 6 apps included interventions of 6 weeks or longer. Common study limitations were small sample sizes; risk of bias; lack of relevant details; and, in some cases, poorly balanced comparison conditions. Further research is required. Evidence for the effectiveness of apps targeting problematic substance use is not compelling, although heterogeneity and trial designs across studies limit the ability to compare efficacy.	Review was relatively recent but had high RoB. Some of the included studies were outside the age range of our review, but seven of 20 studies appeared to be relevant. Authors identified several apps reporting superior outcomes compared to controls but the heterogeneity of the interventions mean its difficult to see reliable conclusions.

AUDIT, alcohol use disorders identification test; CDI, computer delivered intervention; CI, confidence interval; MD, mean difference; OR, odds ratio; RCT, randomised controlled trial; RoB, risk of bias; SMD, standardised mean difference; SMS, short messaging service; WHO, World Health Organization.

^a^ This may represent either the total number of included studies in reviews, or the number of studies included in any particular subgroup, sub population, or outcome of a review that was reported separately and appeared relevant to the aims of this review.

^b^ Emphasis was put on recent reviews, reviews of higher quality based on ROBIS scores and reviews where meta-analysis was conducted. Where reviews carried out a relevant meta-analysis, the pooled results were included. Conclusions from qualitative and/or older reviews were briefly summarised in narrative. Given the rapidly developing technology that exists, reviews were considered as possibly out-of-date if they had a latest search date before 2016 as they were unlikely to represent digital technology that is current, widely used, or advanced enough to have optimal interactivity and features. However, where other evidence was limited these older reviews were included and variously introduced.

Reviews were grouped by type of included participants. College and university students [*n* = 7 reviews (19–25)] as well as participants in school-aged [*n* = 2 reviews (18, 43)]. Two reviews focused particularly on those over 18 years (young adults) so are discussed together (26, 27). The remaining reviews [*n* = 16 (13, 28–42)] covered both adolescents and young adults (variously defined) and were delivered in a community setting. Where a review was not exclusively concerned with students in either college or school, this was grouped as both adolescent and young adult.

For study selection process see PRISMA flow chart in Figure 1.

### Rob assessment

Twenty two of the 27 included reviews had a high RoB, with a low RoB rating in only five of the included reviews (26, 28, 29, 40, 43). Sixteen of the 27 reviews were considered likely to be out-of-date (having a search end date before 2016 and thus likely to detail technology that has been surpassed by newer developments or superseded primary research) (18–22, 26, 29–31, 34–39, 42) and will therefore be unlikely to represent digital technology that is current, widely used, or advanced enough to have optimal interactivity and features (see Supplementary File S3, RoB assessments). However, they have been variously introduced and described for purposes of overview.

### Cancer incidence and adverse events

No reviews identified any cancer related outcomes or reported any adverse events.

### School-aged children

Two reviews restricted to RCTs, both conducted by Champion and colleagues, assessed digital interventions solely in school-aged children. The earlier review by Champion was deemed out of date (pre 2016) with a high RoB and concluded that existing computer- and internet-based prevention programs in schools had the potential to reduce alcohol and other drug use as well as intentions to use substances in the future (18). However, Champion et al. (2019) was higher quality, more up to date and included a meta-analysis. They found that, overall, eHealth school-based multiple health behaviour change interventions were not effective in preventing or reducing alcohol use. This conclusion was based on six studies reporting alcohol use outcomes. Two studies, with four intervention groups, were combined in the meta-analysis and pooled results showed that interventions had no effect on the prevalence of alcohol use (OR = 1.13, 95% CI: 0.95–1.36).

### College/university students

Seven reviews were solely in college or university students (19–25). None were rated at low RoB. All covered a range of interventions (Table 1). Two systematic reviews were judged as out-of-date (19, 20) (Elliott 2008 had searches ending in August 2007 and Carey 2009 did not state the search end date). Of the remaining five, two covered any digital intervention (24, 25) and three focused on internet interventions (21–23). None of the internet intervention reviews included a meta-analysis. Of the two reviews examining any digital interventions, only Prosser and colleagues conducted a meta-analysis (25).

#### Internet intervention

All three internet intervention reviews had some positive conclusions. Bhochhibhoya and colleagues concluded that using the internet as a brief intervention approach can effectively support efforts to reduce binge drinking among college students (21). Leeman and colleagues concluded that there was some evidence supporting very-brief, web-based interventions in reducing alcohol use but not related problems such as increased likelihood of poor academic performance, motor accidents, violence or risky sexual behaviour in college students (22). Bedendo and colleagues presented the most up-to-date review and included the most individual studies of all reviews (23). They concluded that personalised normative feedback and the AlcoholEdu website, the most frequently evaluated interventions among the included studies, were effective in reducing alcohol use in university students. While these reviews do suggest encouraging results, two were out of date and the latest review by Bedenedo (23) did not conduct searches beyond February 2016, so any up to date primary research has not been included in their review. Furthermore, only Leemen and Bedendo utilised RCT evidence, while Bhochihibhoya and colleagues did not clarify included study designs.

#### Any digital intervention

Of the two reviews considering a range of digital intervention type, Dick and colleagues focused on the effectiveness of digital interventions to reduce harm from illicit substance misuse without alcohol use being a focus of the studies in the reviews (24). However, they included four studies in their review that had alcohol outcomes. Their overall conclusions did not relate to alcohol and they did not present a meta-analysis. However, Prosser and colleagues (25) evaluated the effectiveness and moderators of E-interventions vs. assessment only controls in the reduction of alcoholic drinks per week in university students. They included only RCTs. Included studies in this review took place in the United Kingdom (UK), the United States of America (USA), Canada, Netherlands and Sweden. However, most studies took place in the USA. Most, but not all, of the included studies consisted of web-based personalised feedback. Twenty-three studies were included in meta-analyses. E-interventions reduced the number of drinks per week compared to assessment only controls although the overall effect was small (SMD: −0.15, 95% CI: −0.21 to −0.09). Sub-group analysis of web-based personalised feedback interventions demonstrated a small to medium effect on reducing drinks (SMD: −0.19, 95% CI: −0.27 to −0.11). For the other interventions, there was little effect (SMD: −0.07, 95% CI: −0.14 to 0.00). Six studies were included in a further analysis with their included follow-ups ranging from 6 to 12 months' post-intervention. No difference between the groups was found (SMD: −0.05, 95% CI: −0.12 to 0.02). While this would suggest that digital interventions can be useful, and feedback-based interventions are to be noted, this review had searches dating only up to June 2017, so the most recent literature on digital interventions for alcohol for college /university students has not been evaluated. Additionally, this review, like the other reviews in this section, had limitations and was rated as high RoB (Supplementary File S3) (25).

### Young adults only

Two reviews were identified where the target populations could be categorised as young adults, but were not explicitly college or university students, and included studies where participants were generally over the age of 18 years. Two reviews focused particularly on those over 18 years so we have grouped them together (26, 27). The review by Khadjesari and colleagues covered computer-based interventions and was rated at low RoB (26) and the review by O'Rourke and colleagues covered a range of digital interventions, but was rated at high RoB (27). Only the review by Khadjesari and colleagues included a meta-analysis (26).

O'Rourke and colleagues focused on young adults aged 18 to 25 years who were screened as being hazardous drinkers although not receiving specialist services (27). The authors of this qualitative review concluded that the ability to provide personalised electronic feedback resulted in a reduction in alcohol consumption, frequency of binge drinking, and drinking in a non-risky way. However, intervention length did not appear to have an impact on overall effectiveness. This review had searches dating only up to January 2016, so the most recent literature on digital interventions for young adults has again not been evaluated. Additionally, this review had several methodological limitations and was rated at high RoB.

The review by Khadjesari and colleagues included 18 studies of college students, three studies of adult problem drinkers from the general population, two of work-place employees and one of emergency department (ED) attendees (26). Eight studies appeared to screen for hazardous drinking, but the other studies used either a lower cut-off score or did not restrict inclusion based on alcohol intake. Most studies compared a computer-based intervention with a minimally active comparator group. The meta-analyses suggested that computer-based interventions were more effective than minimally active comparator groups (e.g., assessment-only) at reducing grams of alcohol consumed per week in student [mean difference (MD): −19.42, 95% CI: −29.83 to −9.00] and non-student populations (MD: −114.94, 95% CI: −198.6 to −31.29). However, a sensitivity analysis of those studies focusing on more methodologically robust studies showed no difference between intervention and minimally active comparator groups in alcohol consumed per week by students (MD & 95% CI not reported). Few studies investigated non-student populations or compared interventions with active comparator groups. The review only covered studies up to December 2008 and was therefore out-of-date and cannot reflect more recent developments in digital technology.

### Both adolescents and young adults

Sixteen reviews included studies of both adolescents and young adults (variously defined) Three were rated at low RoB (28, 29, 40). Five reviews restricted inclusion to RCTs (28, 30, 31, 34, 38). Four accepted a range of study types (33, 35, 39, 40). However, six reviews did not clarify the type of studies to be included (13, 29, 36, 37, 41, 42). Just four conducted a meta-analysis of included alcohol studies (28, 31, 32, 40). Ten reviews were judged as out-of-date (having a search end date before 2016) (29–31, 34–39, 42).

#### Mobile phone interventions

Two reviews focused solely on mobile phone interventions (32, 33).

Bastola and colleagues' review focused on college students and university students (but defined young adults as under 39 years) (32). The intervention was mobile phone-based text messages as a preventive intervention for problem drinking. The authors commented that message size and frequency varied widely between the studies with reported frequencies ranging from twice weekly to four to six times daily. Seven studies were included of which two reported on longer-term outcomes (six months or more). In the short-term, mobile phone text messages did not reduce the number of drinks per occasion (SMD: 0.28, 95% CI: −0.02 to 0.58) and consumption of average standard glasses per week (SMD: −0.05, 95% CI: −0.15 to 0.05) whereas the risk of binge drinking was significantly higher (OR = 2.45, 95% CI: 1.32–4.53) in the intervention group. Longer-term effects were similar, favouring controls for reduction of binge drinking (OR = 7.24, 95% CI: 2.71–19.31). The authors concluded that text message-based interventions might not be effective in decreasing alcohol intake in this population. They advised further study in particular to determine whether the messages have possible negative effects.

Staiger and colleagues investigated reduction in the use of illicit drugs and tobacco in addition to alcohol (33). Additionally, some of the studies in their systematic review were outside the age range of our review, but seven of 20 studies appeared to be relevant from the study characteristics table. The intervention was mobile apps. Most apps were stand alone, but others had additional components such as supportive counselling or a high-risk patient locator, which sends an alert to patients if they are approaching a high-risk drinking location. The authors identified a number of apps reporting superior outcomes compared to controls including A-CHESS, TeleCoach and CampusGANDR. One app (LBMI-A) reported intervention effects during treatment but not post treatment. There were few commonalities across the more successful apps which varied substantially in intervention length, content, and complexity. However, interestingly they tended to include normative or personalised feedback efficacy and tended to be longer than four weeks in duration. The authors did not find the evidence compelling and advised further research.

#### Computer interventions

One review by Rooke and colleagues focused on computer-based interventions only (30). The review included studies with variously delivered interventions, including those with and without feedback, accessed at home or not, in both offline and online format, and with or without therapist involvement. The effects of computer-based interventions on alcohol were investigated by moderator analysis. Computer-based interventions were effective at reducing alcohol but effects as reported by standardised differences were small (*d* = 0.22, 95% CI: 0.14–0.29) (30). The review, however, was considerably out of date with final searches being January 2009 and was of lower quality.

#### Internet interventions

Four reviews focused on internet interventions for alcohol consumption in both adolescents and young adults (29, 34, 37, 38). None conducted a meta-analysis. Only the review by Bewick and colleagues was rated at low RoB but this review was out-of-date (search end date May 2006) meaning that it did not reflect any of the latest technological developments and no meta-analysis was conducted, instead reporting narratively (29) They report that: “Where web-based personalized feedback alone was compared to web-based feedback combined with additional self-help material the results favoured the combined intervention. Where web-based newsletters with no personalized component were compared to traditional print newsletters the results suggest that traditional print modes of delivery are more effective. However, when a web-based text education website without personalized feedback was compared to a personalized interactive website the results did not favour either intervention.” The reviews by Tait 2010 (34) and White 2010 (38) were also out-of-date (search end dates February 2009 and December 2009, respectively). The review by Giroux and colleagues only included three studies in the age group of interest for alcohol and did not have specific conclusions for this age group (37).

#### Any digital interventions

Nine reviews considered a range of digital interventions for adolescents and young adults (13, 28, 31, 35, 36, 39–42). The evidence was limited again, with outdated research and was mostly high risk of bias as well as a range of different intervention types.

Four reviews were relatively out-of-date, provided only a qualitative synthesis and were at high RoB (35, 36, 39, 42). The focus of the review by Shingleton was on technology-delivered adaptations of motivational interviewing (TAMI) for a range of health-related behaviours (42). Just four of 28 studies in this review were relevant to alcohol consumption in adolescents and young adults. Overall conclusions suggested the feasibility of this type of intervention and the need for further research to better characterise the components of TAMIs. The review by Haug and colleagues was one of the oldest reviews (search end date August 2009) (35). Further research was suggested to test the efficacy of web-based social norms interventions to decrease alcohol consumption in student and non-student samples. The review by Ohinmaa and colleagues was also one of the oldest reviews (search end date May 2009) (39). The focus was on telehealth for a range of substance abuse and addictive behaviours. This review concluded that there are promising studies in internet applications for alcohol addiction when more developed interactive programs are used in motivated high risk/problem drinking populations. The focus of the review by Tebb and colleagues was on how computer-based interventions integrate theories of behaviour change to address alcohol use among adolescents and young adults (36). Whilst this review had a number of methodological limitations and was not current, their conclusion on the need for greater emphasis on the selection and application of theory in computer-based interventions appears appropriate.

Two reviews were more up-to-date, provided a qualitative synthesis, but were at high RoB (13, 41). Firstly, Hutton and colleagues focused on adolescents and young adults (12–26 years) without alcohol dependency or a pre-existing condition related to alcohol and investigated mHealth (social networking sites, SMS and mobile phone applications) (13). Eighteen studies were included and interventions varied in design, participant characteristics, settings, length and outcome measures. Ten studies reported some effectiveness related to interventions with nine reporting a reduction in alcohol consumption. The authors concluded that use of mHealth, particularly text messaging was found to be an acceptable, affordable, and effective way to deliver messages about reducing alcohol consumption to adolescents and young adults. However, they recommended further research using adequately powered sample sizes in varied settings, with adequate periods of intervention and follow-up and underpinned by theoretical perspectives of alcohol consumption. Secondly, Calverley and colleagues included 70 studies investigating alcohol education programmes for adolescents and young adults, 37 were delivered digitally (41). This review assessed the quality of the interventions provided using ten criteria including: based on theoretical framework/s, culturally and context sensitive content, comprehensive interactive training for programme providers, interactive approach to delivery, multi-component approach to delivery skills training to build resilience, accurate content about peer behaviours and social norms, developmentally appropriate information for the target age group and provided resources to reinforce content. The authors concluded that some education programmes have the capacity to positively change alcohol-related behaviour; however, outcome consistency varied even in high-quality programmes.

One review conducted a meta-analysis, but was rated at high RoB and was out-of-date (search end date 25 March 2015) (31). Dedert and colleagues aimed to characterise treatment intensity and systematically review the evidence for efficacy of e-interventions, relative to controls, for reducing alcohol consumption and alcohol-related impairment in adults and college students. E-interventions could be delivered by CD-ROM, online, mobile applications, or interactive voice response. Thirteen of 28 studies were relevant to the age group of our review. The meta-analysis relating to adolescents and young adults was in college students where e-interventions were associated with a small reduction in alcohol consumption at six-month follow-up [MD: −11.7 grams per week (95% CI: −19.3 to −4.1)]. In five trials that used 12-month follow-up assessments analyses revealed no reduction in alcohol consumption [MD: −4.7 grams per week (95% CI: −24.5 to 15.1)]. The authors suggested that future e-interventions could provide more intensive treatment and possibly human support to assist persons in meeting recommended drinking limits (31).

Two reviews were rated at low RoB (28, 40). Both conducted a meta-analysis and were relatively current (search end dates March 2017 and April 2016 respectively).

The high-quality Cochrane review by Kaner and colleagues was on personalised digital interventions for reducing hazardous and harmful alcohol consumption in community-dwelling populations (28). All participants had been screened as risky drinkers. This review gave a clear definition of digital interventions “delivered primarily through a programmable computer or mobile device (laptop, phone or tablet), and were responsive to user input to generate personalised content which aimed to change the participants” alcohol-related behaviours. Interventions were not restricted to those accessible online.' As the focus of the review was not restricted to adolescents and young adults, relevant results are more limited. There were 27 trials with 13,477 participants who were solely adolescents, young adults or college students. The age limits varied, but the maximum specified age in this subgroup of trials was 29 years. One analysis separated trials of younger people and trials of adults using the longest period of follow-up. For adolescents or young adults, the difference between the digital intervention and no or minimal intervention arms in the quantity of alcohol consumed was smaller in magnitude than in the main analysis of adolescents-young adults and adults combined [−13.4 g/week, 95% CI: −19.3 to −7.6 vs. −22.84 g/week (−30.31, −15.36)]. Furthermore, this value differed significantly from the corresponding value based on 14 trials in 5,764 adults (aged >18 years) (−56.1 g/week, 95% CI: −82.1 to −30.0). However, trials of adults were more heterogeneous. Other important conclusions were made regarding the whole population of the review. They stated that low-quality evidence suggested there may be little or no difference in impact on alcohol consumption between digital and face-to-face interventions. They noted that the behaviour change techniques of behaviour substitution, problem solving and credible source were associated with the effectiveness of digital interventions to reduce alcohol consumption and warranted further research. The authors noted that reporting of theory use was very limited and often unclear. Over half of the interventions made no reference to any theories.

The well conducted review by Smedslund and colleagues assessed the effects of early, computerised brief interventions on adolescents and young adults aged 15 to 25 who were high or risky consumers of alcohol and/or cannabis (40). This review included 52 RCTs and quasi-RCTs relevant to alcohol consumption in the target population and performed a series of meta-analyses. Studies in this review were assessed for quality and evidence evaluated using GRADE (44).

Brief interventions in the review by Smedslund and colleagues were defined as “any preventive or therapeutic activity (delivered by a health worker, psychologist, social worker, or volunteer worker) given within a maximum of four structured therapy sessions, each of short duration that lasts between five and ten minutes with a maximum total time of one hour” (40). Eligible comparator conditions were an alternative early, brief intervention, no intervention or waiting list control. The authors commented that most studies were from the USA and targeted high and risky alcohol use among university students. The mode of delivery of most interventions was through a webpage (*n* = 47), while fewer studies used other modes of delivery such as telephone (*n* = 1), CD-ROM (*n* = 2), e-mail (*n* = 3), offline tablet computer (*n* = 1), smartphone app (*n* = 1), text messages (*n* = 3), Facebook (*n* = 1), and chat program (*n* = 1). Results were presented for a range of comparisons including: assessment and feedback vs. no intervention, assessment and feedback vs. assessment only, assessment and feedback vs. and comparison between two types of active interventions.

In a meta-analysis of 15 studies Smedslund and colleagues found that assessment and feedback reduced short-term alcohol consumption compared to no intervention (40). The effect size was small (SMD: −0.17, 95% CI: −0.27 to −0.08) and the quality of the evidence was low. For long-term alcohol consumption three studies showed a similarly small effect size (SMD: −0.17, 95% CI: −0.30 to −0.04). Again, the quality of the evidence was low. Smedslund and colleagues conducted a meta-analysis of 24 studies which showed a small effect size in favour of computerised assessment and feedback vs. assessment only (SMD: −0.15, 95% CI: −0.25 to −0.06). The quality of the evidence was low. For the long-term follow-up there were only three studies, and there was no difference between approaches (SMD: −0.03, 95% CI: −0.19 to 0.12). Similarly, a meta-analysis of seven studies showed no short-term effect of assessment and feedback compared to education (SMD: −0.02, 95% CI: −0.21 to 0.17). The evidence was of very low quality. A meta-analysis of six studies did not find that the short-term effect of computerised brief interventions was different from a brief intervention delivered by a counsellor (SMD: −0.10, 95% CI: −0.30 to 0.11). However, this was based on very low quality evidence. The two studies with long-term effects also showed no difference between approaches (SMD: −0.11, 95% CI: −0.53 to 0.32 (very low quality evidence). A meta-analysis of four studies by the same first author found a 16% short-term reduction in drinking after a repeated assessment and feedback compared to a single assessment and feedback (Rate ratio: 0.84, 95% CI: 0.78 to 0.91). The quality of evidence was graded moderate. Overall conclusions by Smedslund and colleagues were that the interventions reduced alcohol consumption in the short-term compared to no intervention, but the effect size was small, and there was no effect in the long-term.

## Discussion

This review aimed to summarise the evidence for the effectiveness of digital interventions on alcohol consumption in adolescents and young people through a review of systematic reviews. We systematically identified and assessed all relevant evidence and provided a commentary and overview on reported data where possible.

No evidence was identified to demonstrate that digital interventions can reduce cancer incidence in young people through moderation of alcohol consumption. This is unsurprising given that such advances in digital technology are relatively recent, the populations of interest were younger people where cancer incidence is generally lower, and such research would require long term follow up. However, the potential health and social benefits of moderating alcohol consumption are considerable. No reviews reported any adverse events concerned with the use of interventions.

With regards to alcohol consumption outcomes, results were derived from a wide range of studies with considerable heterogeneity and were mostly lower quality. This made it difficult to define any consistent findings that suggest any clear effect. Interventions were variously described and could often fit into more than one category, so we tried to group reviews as closely as possible based on the definitions and descriptions that were provided. Some evidence from systematic reviews exists for those interventions defined as “any digital intervention” in both adolescents and young adults (13, 28, 31, 35, 36, 39–42), internet interventions in both adolescents and young adults (29, 34, 37, 38), computer interventions in young adults only (26) and any digital interventions in school-aged children (18, 43). Minimal evidence exists for college/university students and internet interventions (21–23).

However, the evidence does indicate that some interventions, such as personalised feedback interventions for instance, can potentially have an impact on alcohol consumption in young adults, however, this effect is modest and not conclusive.

The review identified relevant and important shortcomings that should be addressed and are key to designing further research and developing future public health recommendations. These can be defined as two separate but related categories, (1) effectiveness (do results demonstrate the effectiveness of the interventions?) and (2) quality and methodological limitations (is there likely to be sufficient quality and consistency in the data/methods for the effect to be reliable?).

With only 27 reviews identified that fulfilled our criteria the systematic review evidence is overall limited. The definitions of population and intervention that were used by each review varied considerably so that results could not be easily grouped or considered together as they were often essentially comparing different interventions, with different methods on different groups of people. For example, Champion and colleagues (43), included studies which compared school-based digital prevention programmes vs. a control group of no intervention, education as usual, or an alternate evidence-based intervention not delivered via eHealth. However, the range of interventions that were included encompassed various methods of delivery including the internet, computers, tablets, mobile technology, or tele-health. For this reason, we have categorised this review as being “any digital”, as it could not strictly be defined as a more specific intervention where a particular definition can be used such as “mobile” or “computer”. Even within specific intervention definitions, it was not possible to reliably consider sets of data together. Bastola (32) and Staiger (33) for example both considered the effect of mobile phone-based interventions in participants that we defined as “adolescents and young adults”. However, marked differences were evident when examining the characteristics of each review. While Bastola defined young adults as being under 39 years, Staiger's review did not specify any age-related criterion. Bastola and colleagues included text message-based interventions but noted that that there was wide inconsistency in the format of text-based interventions amongst the included studies. Staiger and colleagues, however, included mobile phone app-based interventions, which may have a completely different interactivity to text messaging, that included several distinct apps which were also markedly different from each other in terms of intervention length, content, and complexity. Ultimately, both authors advised that further research was recommended.

In commenting on our findings, it must again be emphasised to the reader that unclear and overlapping definitions should be considered in the interpretation of results. The outcomes used in this review of reviews are those that were chosen in the included systematic reviews, that in turn had to deal with the various definitions in the primary studies. This limited our options to use a comprehensive, clearly defined, and consistent set of outcomes and thereby meant that full systematic groupings and further meta-analysis was not possible.

### Strengths and limitations

Our review was developed using evidence from systematic reviews. Its strengths include comprehensive literature searches without language restriction and across a range of databases and resources and the inclusion of the highest certainty evidence.

Several problems were identified with the included systematic reviews. A number of reviews were out-of-date, which is a highly problematic in a rapidly changing technology field such as digital interventions. If systematic reviews are out-of-date the resulting review of reviews does not include the most recent evidence either. Most reviews were at high risk of bias suggesting that their results and conclusions may not be reliable. In addition, many reviews included digital interventions which were defined in various ways by the authors. There was a high heterogeneity across the reviews in terms of populations, duration of interventions, content and personalisation, comparators and outcomes. Relatively up-to-date and good quality reviews were scarce. While we conducted this process with rigour, there is always the potential that certain evidence was missed, however, we consider that to be of low likelihood and unlikely to have any major impact on the general observations of this review.

**Figure 2:**
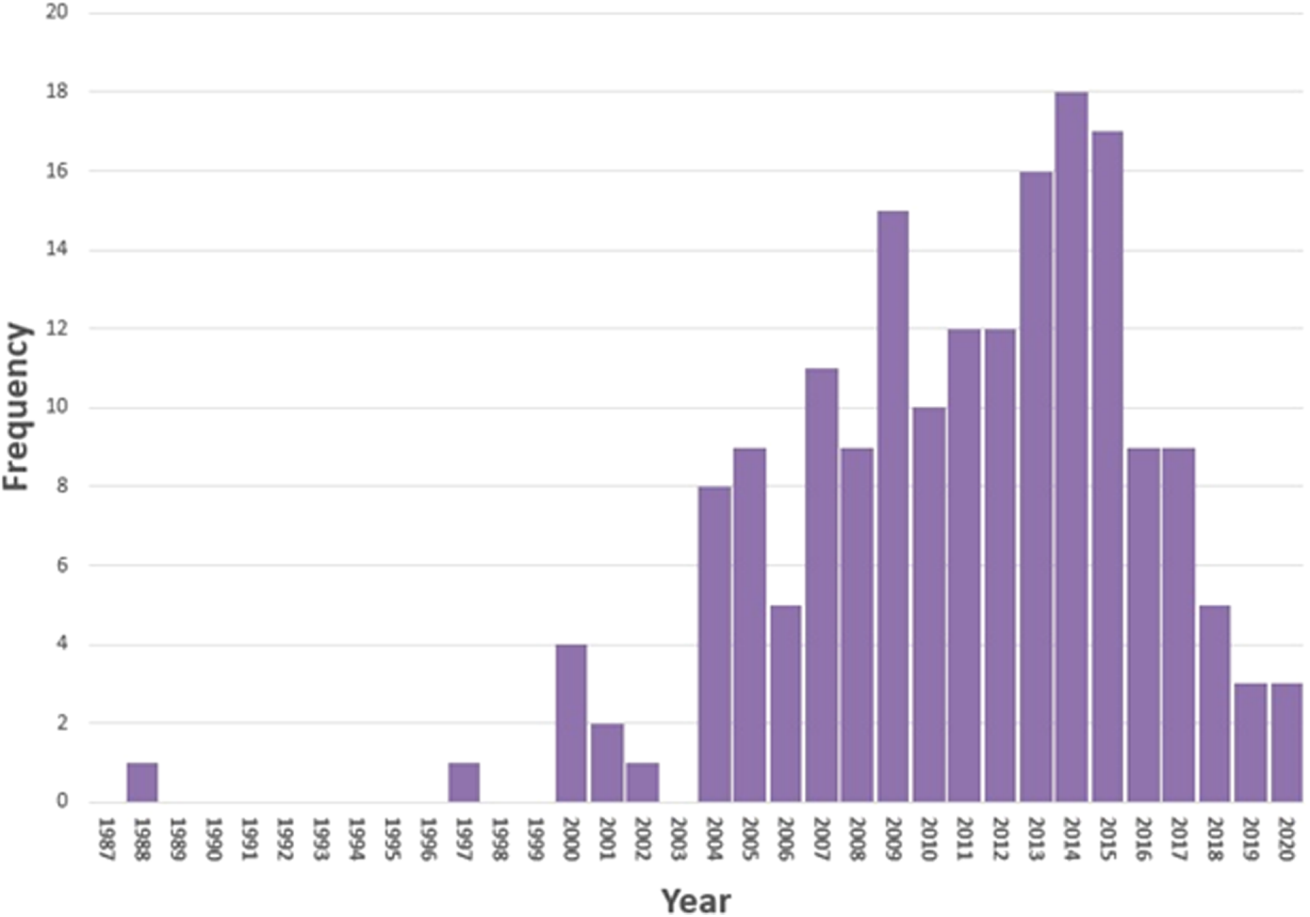
Primary studies identified by the systematic reviews suggests a decline up to 2020, emphasising that any recent literature had not been rigorously reviewed.

The review highlights a decline in primary study numbers included in the systematic reviews up to 2020 (Figure 2) suggesting that any recent literature had not been rigorously reviewed. As can be seen, primary studies included in the systematic reviews peak in 2014. Older reviews will obviously not include more recently published primary studies. Moreover, reviews varied in the inclusion criteria and the numbers of included studies. Thus, there is no certainty that all relevant studies were captured by included systematic reviews and so it is feasible that there may be relevant primary research that has not been identified.

## Conclusions

This review examined existing systematic review literature on digital interventions to reduce alcohol consumption in young people and assess the body of evidence. There is limited systematic review evidence indicating that certain digital interventions may be effective in achieving some positive impact on alcohol consumption in certain groups of young people. However, the observed effect is often small or diminishes when only methodologically robust evidence is considered.

It appears that interventions which offer feedback may be useful approaches for future public health interventions. Future research is necessary that takes a more specific approach, to also address what may be more relevant variables in moderating the impact of an effect, such as feedback vs. non-feedback, short term vs. long term, mobile internet via app vs. computer internet via website. This is an important point, as definitions such as “computer”, “mobile phone” or “digital” are generic and within them are a range of specific “treatments” delivered with specific protocols. It is akin to grouping a range of different analgesics, each with different designs and mechanisms. Given the rapid evolution of such digital technology and the wide variability within interventions, these future efforts may be helpful to elucidate the optimal digital strategy. While this was beyond the scope of this review, the prevalence and potential impact of excessive drinking in younger populations, the current limitations in evidence, and the continually developing potential of digital technology, are key reasons to facilitate further research. The optimal use of digital technology may have the potential to help reduce risky behaviours in young people that may contribute to health harms including cancer.

## Data availability statement

The original contributions presented in the study are included in the article/**Supplementary Material**, further inquiries can be directed to the corresponding author.

## Author contributions

The scope for the rapid review was conceptualized by a task group from Cancer Prevention Europe (CPE), with members LB, CE, JF, KS, JS, MAT, and MW. The review was carried out by KTM, CN, RW, and JK, who also drafted the first version of the manuscript. All authors contributed to the article and approved the submitted version.
