# Supplementary material for: Digital interventions to moderate alcohol consumption in young people: a Cancer Prevention Europe overview of systematic reviews
Source: Front Digit Health. 2023 May 23;5:1178407. doi: 10.3389/fdgth.2023.1178407 (PMC10243367; doi:10.3389/fdgth.2023.1178407)
Supplement: Supplementary file 2 [file Datasheet2.docx]

**Supplementary file 2. Excluded studies.**

Please be aware that this represents the excluded studies from the complete research project as described in the main article and detailed in the PRISMA flow chart (figure 1). This data therefore represent all studies that were excluded after being identified through our wide-ranging search strategy based around several lifestyle factors, alcohol consumption being just one of these. Excluded papers listed here therefore include those that were relevant to other lifestyle factors, as well as those that were alcohol specific. We have included the whole strategy because all aspects of the strategy were relevant in identifying articles specific to this particular review.

**STAGE 1 (Primarily systematic reviews for alcohol, and other lifestyle factors not reported in this review)**

| **Reason for exclusion** | **Number of papers** |
| --- | --- |
| Not a relevant population | 14 |
| Not relevant intervention | 14 |
| Not a relevant comparison | 2 |
| No relevant outcome | 2 |
| Not relevant study design | 4 |
| Duplicate | 1 |
| Unobtainable | 2 |
| Redundant | 5 |
| **TOTAL** | **44** |

| **Author (year)** | **Reason for exclusion** |
| --- | --- |
| Balas (1997)[1] | Not a relevant population |
| Riper (2011)[2] |  |
| Danielsson (2014)[3] |  |
| Durl (2018)[4] |  |
| Stead (2019)[5] |  |
| Dedert (2015)[6] |  |
| Ammerman (2001)[7] |  |
| Niu (2016)[8] |  |
| Rhodes (2020)[9] |  |
| Wieland (2012)[10] |  |
| Jenkins (2009)[11] |  |
| Lee (2016)[12] |  |
| Gal (2018)[13] |  |
| Thomas Craig (2020)[14] |  |
| Wachtel (2010)[15] | Not relevant intervention |
| Tancred (2019)[16] |  |
| Tanner-Smith (2015)[17] |  |
| Tanner-Smith (2016)[18] |  |
| Foxcroft (2012)[19] |  |
| Foxcroft (2012)[20] |  |
| Tamony (2015)[21] |  |
| Lefio (2013)[22] |  |
| Hale (2014)[23] |  |
| Jepson (2010)[24] |  |
| Brown (2019)[25] |  |
| Brannon (2015)[26] |  |
| Chaplais (2015)[27] |  |
| Klos (2020)[28] |  |
| Yonker (2015)[29] | Not a relevant comparison |
| Zapata-Lamana (2020)[30] |  |
| Rodriguez (2014)[31] | No relevant outcome |
| Das (2016)[32] |  |
| Laging (2012)[33] | Not relevant study design |
| Whatnall (2019)[34] |  |
| Kaiser (2009)[35] |  |
| Denison (2010)[36] |  |
| Bedendo (2018)[37] | Duplicate |
| Su (2014)[38] | Unobtainable |
| Kyung (2018)[39] |  |
| Zisserson (2007)[40] | Redundant |
| Mason (2015)[41] |  |
| Dedert (2014)[42] |  |
| Kazemi (2017)[43] |  |
| Badawy (2017)[44] |  |

**STAGE 2 (Primarily primary studies and additional review searches for alcohol, and other lifestyle factors not reported in this review)**

| **Reason for exclusion** | **Number of papers** |
| --- | --- |
| Not a relevant population | 82 |
| Not relevant intervention | 34 |
| Not a relevant comparison | 8 |
| No relevant outcome | 21 |
| Not relevant study design | 9 |
| Duplicate | 5 |
| Unobtainable | 25 |
| Redundant review/primary studies | 39 |
| Protocols only | 38 |
| **TOTAL** | **261** |

| **Author (year)** | **Reason for exclusion** |
| --- | --- |
| Kerr 2016[45] | Not a relevant population |
| Thompson 2015[46] |  |
| Wang 2016[47] |  |
| O'Brien 2016[48] |  |
| Shoneye 2019[49] |  |
| Boumparis 2021[50] |  |
| Fucito 2021[51] |  |
| Fodor 2020[52] |  |
| Nordholt 2020[53] |  |
| Mujcic 2020[54] |  |
| Hadjistavropoulos 2020[55] |  |
| Bertholet 2020[56] |  |
| Jo 2019[57] |  |
| Norman 2019[58] |  |
| Sanchez 2018[59] |  |
| Bertholet 2018[60] |  |
| Guillemont 2017[61] |  |
| Attwood 2017[62] |  |
| Pedersen 2017[63] |  |
| Frohlich 2018[64] |  |
| Thomas 2018[65] |  |
| Hutchesson 2018[66] |  |
| Duroy 2016[67] |  |
| Pedersen 2016[68] |  |
| Kvillemo 2020[69] |  |
| Sharpe 2018[70] |  |
| Cunningham 2018[71] |  |
| Omar 2021[72] |  |
| Berg 2020[73] |  |
| Reddy 2021[74] |  |
| Lampousi 2020[75] |  |
| Holmes 2020[76] |  |
| Ramalho 2020[77] |  |
| Andrade 2020[78] |  |
| Mack 2020[79] |  |
| Coknaz 2019[80] |  |
| Ye 2019[81] |  |
| Lonsdale 2019[82] |  |
| Staiano 2018[83] |  |
| Fu 2018[84] |  |
| Bruno 2018[85] |  |
| Glapa 2018[86] |  |
| Plotnikoff 2017[87] |  |
| Kovalskys 2017[88] |  |
| Shin 2017[89] |  |
| Partridge 2017[90] |  |
| Armbrust 2017[91] |  |
| Mehdizadeh 2018[92] |  |
| Kebede 2018[93] |  |
| Simons 2018[94] |  |
| Herget 2016[95] |  |
| Hartman 2016[96] |  |
| Zoellner 2016[97] |  |
| Shin 2016[98] |  |
| Lonsdale 2016[99] |  |
| Howie 2016[100] |  |
| Biddle 2015[101] |  |
| Sharma 2015[102] |  |
| Meldrum 2015[103] |  |
| Ptomey 2015[104] |  |
| Fassnacht 2015[105] |  |
| Cayir 2015[106] |  |
| Sun 2016[107] |  |
| Allman-Farinelli 2016[108] |  |
| Zhang 2015[109] |  |
| Partridge 2015[110] |  |
| Zhou 2021[111] |  |
| Allafi 2020[112] |  |
| Mok 2020[113] |  |
| Ulanday 2020[114] |  |
| Salsman 2020[115] |  |
| Likhitweerawong 2020[116] |  |
| Bovi 2019[117] |  |
| Stasinaki 2018[118] |  |
| L'Allemand 2018[119] |  |
| Bos 2017[120] |  |
| Livingstone 2017[121] |  |
| Yoshinaga 2016[122] |  |
| Markert 2015[123] |  |
| Lau 2015[124] |  |
| Hieftje 2013[125] |  |
| Cullen 2005[126] |  |
| Lubans 2009[127] | Not relevant intervention |
| Yang 2017[128] |  |
| Franken 2018[129] |  |
| Teesson 2020[130] |  |
| Neighbors 2019[131] |  |
| Braitman 2016[132] |  |
| Champion 2016[133] |  |
| Deluca 2021[134] |  |
| Newton 2020[135] |  |
| Schwarz 2020[136] |  |
| Meyer 2021[137] |  |
| Cowley 2021[138] |  |
| Corepal 2019[139] |  |
| Lee 2020[140] |  |
| Sabooteh 2021[141] |  |
| Sundgot-Borgen 2020[142] |  |
| Silva 2020[143] |  |
| Zhamardiy 2020[144] |  |
| Memon 2018[145] |  |
| Stephens 2017[146] |  |
| Sharp 2016[147] |  |
| Klausen 2016[148] |  |
| Anson 2016[149] |  |
| Jussila 2015[150] |  |
| Abraham 2015[151] |  |
| Dzielska 2020[152] |  |
| Fijalkowska 2019[153] |  |
| Dabbas 2019[154] |  |
| Buchter 2016[155] |  |
| Morrison 2015[156] |  |
| Da Silva 2019[157] |  |
| Laska 2016[158] |  |
| Collins 2014[159] |  |
| Kattelmann 2014[160] |  |
| Wang 2015[161] | Not a relevant comparison |
| Rodgers 2016[162] |  |
| Folkvord 2020[163] |  |
| Lei 2020[164] |  |
| Hickman 2018[165] |  |
| Januraga 2021[166] |  |
| Sundstrom 2016[167] |  |
| Hwang 2018[168] |  |
| Holzmann 2020[169] | No relevant outcome |
| Chaput 2015[170] |  |
| Ruggiero 2020[171] |  |
| Sanatkar 2021[172] |  |
| Shell 2019[173] |  |
| Gilmore 2018[174] |  |
| Watson 2017[175] |  |
| Walsh 2017[176] |  |
| Bountress 2017[177] |  |
| Helmer 2016[178] |  |
| Bendtsen 2020[179] |  |
| Thornton 2018[180] |  |
| Arman 2019[181] |  |
| Heino 2018[182] |  |
| Liu 2018[183] |  |
| Del Corral 2018[184] |  |
| Schoeppe 2017[185] |  |
| Turner 2015[186] |  |
| Mackey 2015[187] |  |
| Nguyen 2015[188] |  |
| Lao 2011[189] |  |
| Lam 2020[190] | Not relevant study design |
| Teesson 2020[191] |  |
| Champion 2017[192] |  |
| Thomas 2017[193] |  |
| Carreiro 2018[194] |  |
| Wright 2017[195] |  |
| Tebb 2016[196] |  |
| Saucedo-Araujo 2020[197] |  |
| Lee 2019[198] |  |
| Brannon 2015[199] | Duplicate |
| Gao 2020[200] |  |
| Kim 2019[201] |  |
| Ramírez-Granizo 2020[202] |  |
| Bedendo 2018[203] |  |
| Pagkalos 2016[204] | Unobtainable |
| O'Connor 2016[205] |  |
| Lee 2021[206] |  |
| Ahn 2020[207] |  |
| Deluca 2020[208] |  |
| Goncalves 2019[209] |  |
| Bernstein 2019[210] |  |
| Bedendo 2019[211] |  |
| Gardner 2018[212] |  |
| Bedendo 2018[213] |  |
| Matteucci 2018[214] |  |
| Harris 2018[215] |  |
| Chao 2017[216] |  |
| Litt 2017[217] |  |
| Neale 2017[218] |  |
| Bernstein 2017[219] |  |
| DiBello 2017[220] |  |
| Carey 2016[221] |  |
| Cadigan 2016[222] |  |
| Earle 2016[223] |  |
| Gilmore 2016[224] |  |
| Neale 2016[225] |  |
| Gelabert Carulla 2020[226] |  |
| Maselli 2019[227] |  |
| Ptomey 2017[228] |  |
| Gao 2015[229] | Redundant review/primary studies |
| Quelly 2016[230] |  |
| Santos 2021[231] |  |
| Shin 2019[232] |  |
| Williams 2020[233] |  |
| Alcantara 2019[234] |  |
| Goodyear 2021[235] |  |
| De Cock 2018[236] |  |
| Simons 2015[237] |  |
| Lubans 2016[238] |  |
| Diestelkamp 2021[239] |  |
| Bedendo 2020[240] |  |
| Martinez-Montilla 2020[241] |  |
| Bedendo 2019[242] |  |
| Zamboanga 2019[243] |  |
| Braitman 2018[244] |  |
| Bernstein 2018[245] |  |
| Gilbertson 2018[246] |  |
| Davies 2017[247] |  |
| Leeman 2016[248] |  |
| Linowski 2016[249] |  |
| Jander 2016[250] |  |
| Caudwell 2016[251] |  |
| Strohman 2016[252] |  |
| Berman 2016[253] |  |
| Valeriani 2021[254] |  |
| Lee 2021[255] |  |
| Brannon 2018[256] |  |
| Muller 2016[257] |  |
| Garde 2015[258] |  |
| Creaser 2021[259] |  |
| Chung 2017[260] |  |
| Simons 2015[261] |  |
| Militello 2012[262] |  |
| Thompson 2009[263] |  |
| Ezendam 2012[264] |  |
| Smith 2014[265] |  |
| Baranowski 2003[266] |  |
| Whittemore 2013[267] |  |
| Magalhaes 2020[268] | Protocols only |
| Chagas 2018[269] |  |
| Belogianni 2019[270] |  |
| Melo 2020[271] |  |
| Bonar 2020[272] |  |
| Haug 2020[273] |  |
| Pedersen 2019[274] |  |
| Diestelkamp 2019[275] |  |
| Haug 2018[276] |  |
| Lima-Serrano 2018[277] |  |
| Ramo 2018[278] |  |
| Stapinski 2019[279] |  |
| Riordan 2018[280] |  |
| Pedersen 2016[281] |  |
| Ip 2016[282] |  |
| Debenham 2021[283] |  |
| Trottier 2021[284] |  |
| Oppezzo 2020[285] |  |
| Fisher 2020[286] |  |
| Michalsen 2020[287] |  |
| Moore 2020[288] |  |
| Partridge 2020[289] |  |
| Szabo-Reed 2020[290] |  |
| Wang 2021[291] |  |
| Masse 2020[292] |  |
| Puigdomenech 2019[293] |  |
| Ptomey 2019[294] |  |
| Liang 2019[295] |  |
| Podina 2017[296] |  |
| Ridgers 2017[297] |  |
| Jones Bell 2019[298] |  |
| Donnelly 2016[299] |  |
| Lonsdale 2016[300] |  |
| Tzelepis 2015[301] |  |
| Hagger 2015[302] |  |
| Hurley 2015[303] |  |
| Downs 2020[304] |  |
| Middelweerd 2015[305] |  |

1. Balas EA, Jaffrey F, Kuperman GJ, Boren SA, Brown GD, Pinciroli F, et al. Electronic communication with patients: evaluation of distance medicine technology. JAMA. 1997;278(2):152-9.

2. Riper H, Spek V, Boon B, Conijn B, Kramer J, Martin-Abello K, et al. Effectiveness of E-self-help interventions for curbing adult problem drinking: a meta-analysis. J Med Internet Res. 2011;13(2):e42.

3. Danielsson AK, Eriksson AK, Allebeck P. Technology-based support via telephone or web: a systematic review of the effects on smoking, alcohol use and gambling. Addict Behav. 2014;39(12):1846-68.

4. Durl J, Dietrich T, Pang B, Potter L-E, Carter L. Utilising virtual reality in alcohol studies: a systematic review. Health Educ J. 2018;77(2):212-25.

5. Stead M, Angus K, Langley T, Katikireddi SV, Hinds K, Hilton S, et al. Mass media to communicate public health messages in six health topic areas: a systematic review and other reviews of the evidence. Public Health Res. 2019;7:1-238.

6. Dedert EA, McDuffie JR, Stein R, McNiel JM, Kosinski AS, Freiermuth CE, et al. Electronic interventions for alcohol misuse and alcohol use disorders: a systematic review. Ann Intern Med. 2015;163(3):205-14.

7. Database of Abstracts of Reviews of Effects. The efficacy of interventions to modify dietary behavior related to cancer risk. Volume 1: evidence report and appendices 2003 [cited 8.9.21]. Available from: <http://www.crd.york.ac.uk/CRDWeb/ShowRecord.asp?ID=12002008219> Appraisal of: Ammerman A, Lindquist C, Hersey J, Jackman AM, Gavin NI, Garces C, et al. *The efficacy of interventions to modify dietary behavior related to cancer risk. Volume 1: evidence report and appendices. 2001.* Rockville (MD): Agency for Healthcare Research and Quality, 2001.

8. Niu J. A meta-analysis evaluation for the efficacy of internet-based interventions for healthy diet and waist circumference reduction. PhD [Dissertation]. Indianapolis (IN): Indiana University; 2016.

9. Rhodes A, Smith AD, Chadwick P, Croker H, Llewellyn CH. Exclusively digital health interventions targeting diet, physical activity, and weight gain in pregnant women: systematic review and meta-analysis. JMIR Mhealth Uhealth. 2020;8(7):e18255.

10. Wieland LS, Falzon L, Sciamanna CN, Trudeau KJ, Brodney Folse S, Schwartz JE, et al. Interactive computer‐based interventions for weight loss or weight maintenance in overweight or obese people. Cochrane Database Syst Rev. 2012;Issue 8:Art. No.: CD007675.

11. Jenkins A, Christensen H, Walker JG, Dear K. The effectiveness of distance interventions for increasing physical activity: a review. Am J Health Promot. 2009;24(2):102-17.

12. Lee J, Piao M, Byun A, Kim J. A systematic review and meta-analysis of intervention for pediatric obesity using mobile technology. Stud Health Technol Inform. 2016;225:491-4.

13. Gal R, May AM, van Overmeeren EJ, Simons M, Monninkhof EM. The effect of physical activity interventions comprising wearables and smartphone applications on physical activity: a systematic review and meta-analysis. Sports Med Open. 2018;4(1):42.

14. Thomas Craig KJ, Morgan LC, Chen CH, Michie S, Fusco N, Snowdon JL, et al. Systematic review of context-aware digital behavior change interventions to improve health. Transl Behav Med. 2020;11(1037-48).

15. Wachtel T, Staniford M. The effectiveness of brief interventions in the clinical setting in reducing alcohol misuse and binge drinking in adolescents: a critical review of the literature. J Clin Nurs. 2010;19(5-6):605-20.

16. Tancred T, Melendez-Torres G, Paparini S, Fletcher A, Stansfield C, Thomas J, et al. How can interventions integrating health and academic education in schools help prevent substance misuse and violence, and reduce health inequalities among young people? Systematic review and evidence synthesis. Public Health Res [Internet]. September 2019 [cited 19.5.21] 7(17). Available from: <http://www.nets.nihr.ac.uk/projects/phr/145215>.

17. Tanner-Smith EE, Lipsey MW. Brief alcohol interventions for adolescents and young adults: a systematic review and meta-analysis. J Subst Abuse Treat. 2015;51:1-18.

18. Tanner-Smith EE, Risser MD. A meta-analysis of brief alcohol interventions for adolescents and young adults: variability in effects across alcohol measures. Am J Drug Alcohol Abuse. 2016;42(2):140-51.

19. Foxcroft DR, Tsertsvadze A. Universal alcohol misuse prevention programmes for children and adolescents: Cochrane systematic reviews. Perspect Public Health. 2012;132(3):128-34.

20. Foxcroft DR, Tsertsvadze A. Cochrane Review: universal school-based prevention programs for alcohol misuse in young people. Evidence-Based Child Health: A Cochrane Review Journal. 2012;7(2):450-575.

21. Tamony P, Holt R, Barnard K. The role of mobile applications in improving alcohol health literacy in young adults with type 1 diabetes: help or hindrance? J Diabetes Sci Technol. 2015;9(6):1313-20.

22. Lefio LÁ, Villarroel SR, Rebolledo C, Zamorano P, Rivas K. Effective interventions in the problematic use of alcohol and other drugs. Rev panam salud pública. 2013;34(4):257-66.

23. Hale DR, Fitzgerald-Yau N, Viner RM. A systematic review of effective interventions for reducing multiple health risk behaviors in adolescence. Am J Public Health. 2014;104(5):e19-41.

24. Jepson RG, Harris FM, Platt S, Tannahill C. The effectiveness of interventions to change six health behaviours: a review of reviews. BMC Public Health. 2010;10(1):538.

25. Brown T, Moore THM, Hooper L, Gao Y, Zayegh A, Ijaz S, et al. Interventions for preventing obesity in children. Cochrane Database Syst Rev. 2019;Issue 7:Art. No.: CD001871.

26. Brannon EE, Cushing CC. A systematic review: is there an app for that? Translational science of pediatric behavior change for physical activity and dietary interventions. J Pediatr Psychol. 2015;40(4):373-84.

27. Chaplais E, Naughton G, Thivel D, Courteix D, Greene D. Smartphone interventions for weight treatment and behavioral change in pediatric obesity: a systematic review. Telemed J E Health. 2015;21(10):822-30.

28. Klos L, Feil K, Eberhardt T, Jekauc D. Interventions to promote positive affect and physical activity in children, adolescents and young adults-a systematic review. Sports (Basel). 2020;8(2).

29. Yonker LM, Zan S, Scirica CV, Jethwani K, Kinane TB. "Friending" teens: systematic review of social media in adolescent and young adult health care. J Med Internet Res. 2015;17(1):e4.

30. Zapata-Lamana R, Lalanza JF, Losilla JM, Parrado E, Capdevila L. mHealth technology for ecological momentary assessment in physical activity research: a systematic review. Peerj. 2020;8:e8848.

31. Rodriguez DM, Teesson M, Newton NC. A systematic review of computerised serious educational games about alcohol and other drugs for adolescents. Drug Alcohol Rev. 2014;33(2):129-35.

32. Das JK, Salam RA, Arshad A, Finkelstein Y, Bhutta ZA. Interventions for adolescent substance abuse: an overview of systematic reviews. J Adolesc Health. 2016;59(4S):S61-75.

33. Laging M. [Web-based interventions for addressing college drinking]. Sucht. 2012;58(2):85-96.

34. Whatnall M, Patterson A, Hutchesson M. A brief web-based nutrition intervention for young adult university students: development and evaluation protocol using the PRECEDE-PROCEED model. JMIR Res Protoc. 2019;8(3):e11992.

35. Kaiser NC, Owen JE, Winzelberg AJ. Technological advances in modifying adolescent health risk behaviors. In: R. J. DiClemente, J. S. Santelli, R. A. Crosby, editors. Adolescent health: understanding and preventing risk behaviors: Jossey-Bass/Wiley; 2009.

36. Denison E, Underland V, Nilsen ES, Fretheim A. [Effects of interventions outside the health services for increased physical activity among adults]. Oslo: Norwegian Knowledge Centre for the Health Services (NOKC); 2010 [cited 21.5.21].

37. Bedendo A, Monezi Andrade AL, Noto AR. [Internet-based alcohol interventions for college students: systematic review]. Rev Panam Salud Publica. 2018;42:e54.

38. Su MC, Lin CL, Tsao LI. The efficacy of e-health management on weight control in adolescents: a systematic review. J Nurs. 2014;61(1):74-84.

39. Kyung PB. Systematic review on the online adolescent obesity prevention program. J Kyungpook Nurs Sci. 2018;22(1):41-50.

40. Zisserson RN, Palfai T, Saitz R. "No-contact" interventions for unhealthy college drinking: efficacy of alternatives to person-delivered intervention approaches. Subst Abus. 2007;28(4):119-31.

41. Mason M, Ola B, Zaharakis N, Zhang J. Text messaging interventions for adolescent and young adult substance use: a meta-analysis. Prev Sci. 2015;16(2):181-8.

42. Dedert E, Williams JW, Stein R, McNeil JM, McDuffie J, Ross I, et al. e-Interventions for alcohol misuse [Internet]. Washington (DC): Department of Veterans Affairs Health Services Research & Development Service, 2014 [cited 10.9.21]. Available from: <https://www.ncbi.nlm.nih.gov/books/n/vaalcoholmis/pdf/>.

43. Kazemi DM, Borsari B, Levine MJ, Li S, Lamberson KA, Matta LA. A systematic review of the mhealth interventions to prevent alcohol and substance abuse. J Health Commun. 2017;22(5):1-20.

44. Badawy SM, Kuhns LM. Texting and mobile phone app interventions for improving adherence to preventive behavior in adolescents: a systematic review. JMIR Mhealth Uhealth. 2017;5(4):e50.

45. Kerr DA, Harray AJ, Pollard CM, Dhaliwal SS, Delp EJ, Howat PA, et al. The connecting health and technology study: a 6-month randomized controlled trial to improve nutrition behaviours using a mobile food record and text messaging support in young adults. Int J Behav Nutr Phys Act. 2016;13:52.

46. Thompson D, Bhatt R, Vazquez I, Cullen KW, Baranowski J, Baranowski T, et al. Creating action plans in a serious video game increases and maintains child fruit-vegetable intake: a randomized controlled trial. Int J Behav Nutr Phys Act. 2015;12:39.

47. Wang Q, Egelandsdal B, Amdam GV, Almli VL, Oostindjer M. Diet and physical activity apps: perceived effectiveness by app users. JMIR Mhealth Uhealth. 2016;4(2):e33.

48. O'Brien LM, Palfai TP. Efficacy of a brief web-based intervention with and without SMS to enhance healthy eating behaviors among university students. Eat Behav. 2016;23:104-9.

49. Shoneye CL, Dhaliwal SS, Pollard CM, Boushey CJ, Delp EJ, Harray AJ, et al. Image-based dietary assessment and tailored feedback using mobile technology: mediating behavior change in young adults. Nutrients. 2019;11(2):435.

50. Boumparis N, Schulte MH, Kleiboer A, Huizink A, Riper H. A mobile intervention to promote low-risk drinking habits in young adults: protocol for a randomized controlled trial. JMIR Res Protoc. 2021;10(6):e29750.

51. Fucito LM, Ash GI, DeMartini KS, Pittman B, Barnett NP, Li CR, et al. A multimodal mobile sleep intervention for young adults engaged in risky drinking: protocol for a randomized controlled trial. JMIR Res Protoc. 2021;10(2):e26557.

52. Fodor MC, Grekin ER, Beatty JR, McGoron L, Ondersma SJ. Participant satisfaction with computer-delivered intervention components and its relation to alcohol outcomes. Subst Use Misuse. 2020;55(14):2332-40.

53. Nordholt PU, Christalle E, Zill JM, Dirmaier J. Engagement with a web-based intervention to reduce harmful drinking: secondary analysis of a randomized controlled trial. J Med Internet Res. 2020;22(11):e18826.

54. Mujcic A, Linke S, Hamilton F, Phillips A, Khadjesari Z. Engagement with motivational interviewing and cognitive behavioral therapy components of a web-based alcohol intervention, elicitation of change talk and sustain talk, and impact on drinking outcomes: secondary data analysis. J Med Internet Res. 2020;22(9):e17285.

55. Hadjistavropoulos HD, Mehta S, Wilhelms A, Keough MT, Sundstrom C. A systematic review of internet-delivered cognitive behavior therapy for alcohol misuse: study characteristics, program content and outcomes. Cogn Behav Ther. 2020;49(4):327-46.

56. Bertholet N, Schmutz E, Grazioli VS, Faouzi M, McNeely J, Gmel G, et al. Smartphone-based secondary prevention intervention for university students with unhealthy alcohol use identified by screening: study protocol of a parallel group randomized controlled trial. Trials. 2020;21(1):191.

57. Jo SJ, Lee HK, Kang K, Joe KH, Lee SB. Efficacy of aweb-based screening and brief intervention to prevent problematic alcohol use in Korea: results of a randomized controlled trial. Alcohol Clin Exp Res. 2019;43(10):2196-202.

58. Norman P, Webb TL, Millings A, Pechey L. Does the structure (tunneled vs. free-roam) and content (if-then plans vs. choosing strategies) of a brief online alcohol intervention effect engagement and effectiveness? A randomized controlled trial. Transl Behav Med. 2019;9(6):1122-30.

59. Sanchez ZM, Sanudo A. Web-based alcohol intervention for nightclub patrons: opposite effects according to baseline alcohol use disorder classification. Subst Abus. 2018;39(3):361-70.

60. Bertholet N, Studer J, Cunningham JA, Gmel G, Burnand B, Daeppen JB. Four-year follow-up of an internet-based brief intervention for unhealthy alcohol use in young men. Addiction. 2018;113(8):1517-21.

61. Guillemont J, Cogordan C, Nalpas B, Nguyen-Thanh V, Richard JB, Arwidson P. Effectiveness of a web-based intervention to reduce alcohol consumption among French hazardous drinkers: a randomized controlled trial. Health Educ Res. 2017;32(4):332-42.

62. Attwood S, Parke H, Larsen J, Morton KL. Using a mobile health application to reduce alcohol consumption: a mixed-methods evaluation of the drinkaware track & calculate units application. BMC Public Health. 2017;17(1):394.

63. Pedersen ER, Parast L, Marshall GN, Schell TL, Neighbors C. A randomized controlled trial of a web-based, personalized normative feedback alcohol intervention for young-adult veterans. J Consult Clin Psychol. 2017;85(5):459-70.

64. Frohlich JR, Rapinda KK, Schaub MP, Wenger A, Baumgartner C, Johnson EA, et al. Efficacy of an online self-help treatment for comorbid alcohol misuse and emotional problems in young adults: protocol for a randomized controlled trial. JMIR Res Protoc. 2018;7(11):e11298.

65. Thomas K, Mussener U, Linderoth C, Karlsson N, Bendtsen P, Bendtsen M. Effectiveness of a text messaging-based intervention targeting alcohol consumption among university students: randomized controlled trial. JMIR Mhealth Uhealth. 2018;6(6):e146.

66. Hutchesson MJ, Callister R, Morgan PJ, Pranata I, Clarke ED, Skinner G, et al. A targeted and tailored ehealth weight loss program for young women: the Be Positive Be Healthe randomized controlled trial. Healthcare (Basel). 2018;6(2):39.

67. Duroy D, Boutron I, Baron G, Ravaud P, Estellat C, Lejoyeux M. Impact of a computer-assisted screening, brief intervention and referral to treatment on reducing alcohol consumption among patients with hazardous drinking disorder in hospital emergency departments. The randomized BREVALCO trial. Drug Alcohol Depend. 2016;165:236-44.

68. Pedersen ER, Parast L, Marshall GN, Schell TL, Neighbors C. A pilot randomized controlled trial for a web-based personalized normative feedback alcohol intervention for young adult veterans. Presented at the 39th Annual Scientific Meeting of the Research Society on Alcoholism; 25-29 June 2016; New Orleans (LO). Alcohol Clin Exp Res. 2016;40(Suppl 1):68A.

69. Kvillemo P, Strandberg AK, Gripenberg J, Berman AH, Skoglund C, Elgan TH. Effects of an automated digital brief prevention intervention targeting adolescents and young adults with risky alcohol and other substance use: Study protocol for a randomised controlled trial. BMJ OPEN. 2020;10(5):e034894.

70. Sharpe S, Kool B, Whittaker R, Lee AC, Reid P, Civil I, et al. Effect of a text message intervention to reduce hazardous drinking among injured patients discharged from a trauma ward: a randomized controlled trial. npj Digital Medicine. 2018;1(1):13.

71. Cunningham JA, Hendershot CS, Kay-Lambkin F, Neighbors C, Griffiths KM, Bennett K, et al. Does providing a brief internet intervention for hazardous alcohol use to people seeking online help for depression reduce both alcohol use and depression symptoms among participants with these co-occurring disorders? Study protocol for a randomised controlled trial. BMJ OPEN. 2018;8(7):e022412.

72. Omar NO, Ahmad RA, Mohd Shah MS, Aminuddin AA, Chellappan KC. Amelioration of inflammation in young men with cardiovascular risks participating pedometer-based walking programme. Med J Malaysia. 2021;76(3):375-81.

73. Berg S, Forest J, Stenseng F. Whenpassion does not change, but emotions do: testing a social media intervention related to exercise activity engagement. Front Psychol. 2020;11:71.

74. Reddy P, Dukhi N, Sewpaul R, Ellahebokus MAA, Kambaran NS, Jobe W. Mobile health interventions addressing childhood and adolescent obesity in Sub-Saharan Africa and Europe: current landscape and potential for future research. Front Public Health. 2021;9:604439.

75. Lampousi AM, Berglind D, Forsell Y. Association of changes in cardiorespiratory fitness with health-related quality of life in young adults with mobility disability: secondary analysis of a randomized controlled trial of mobile app versus supervised training. BMC Public Health. 2020;20(1):1721.

76. Holmes H, Palacios C, Wu Y, Banna J. Effect of a short message service intervention on excessive gestational weight gain in a low-income population: a randomized controlled trial. Nutrients. 2020;12(5):1428.

77. Ramalho S, Saint-Maurice PF, Silva D, Mansilha HF, Silva C, Goncalves S, et al. APOLO-Teens, a web-based intervention for treatment-seeking adolescents with overweight or obesity: study protocol and baseline characterization of a Portuguese sample. Eat Weight Disord. 2020;25(2):453-63.

78. Andrade A, Cruz WMD, Correia CK, Santos ALG, Bevilacqua GG. Effect of practice exergames on the mood states and self-esteem of elementary school boys and girls during physical education classes: a cluster-randomized controlled natural experiment. PLoS One. 2020;15(6):e0232392.

79. Mack I, Reiband N, Etges C, Eichhorn S, Schaeffeler N, Zurstiege G, et al. The Kids Obesity Prevention Program: cluster randomized controlled trial to evaluate a serious game for the prevention and treatment of childhood obesity. J Med Internet Res. 2020;22(4):e15725.

80. Coknaz D, Mirzeoglu AD, Atasoy HI, Alkoy S, Coknaz H, Goral K. A digital movement in the world of inactive children: favourable outcomes of playing active video games in a pilot randomized trial. Eur J Pediatr. 2019;178(10):1567-76.

81. Ye S, Pope ZC, Lee JE, Gao Z. Effects of school-based exergaming on urban children's physical activity and cardiorespiratory fitness: a quasi-experimental study. Int J Environ Res Public Health. 2019;16(21):4080.

82. Lonsdale C, Lester A, Owen KB, White RL, Peralta L, Kirwan M, et al. An internet-supported school physical activity intervention in low socioeconomic status communities: results from the Activity and Motivation in Physical Education (AMPED) cluster randomised controlled trial. Br J Sports Med. 2019;53(6):341-7.

83. Staiano AE, Beyl RA, Guan W, Hendrick CA, Hsia DS, Newton RL, Jr. Home-based exergaming among children with overweight and obesity: a randomized clinical trial. Pediatr Obes. 2018;13(11):724-33.

84. Fu Y, Burns RD, Constantino N, Zhang P. Differences in step counts, motor competence, and enjoyment between an exergaming group and a non-exergaming group. Games Health J. 2018;7(5):335-40.

85. Bruno A, Escobar P, Cebolla A, Alvarez-Pitti J, Guixeres J, Lurbe E, et al. Home-exercise childhood obesity intervention: a randomized clinical trial comparing print versus web-based (Move It) platforms. J Pediatr Nurs. 2018;42:e79-84.

86. Glapa A, Grzesiak J, Laudanska-Krzeminska I, Chin MK, Edginton CR, Mok MMC, et al. The impact of brain breaks classroom-based physical activities on attitudes toward physical activity in Polish school children in third to fifth grade. Int J Environ Res Public Health. 2018;15(2):368.

87. Plotnikoff RC, Wilczynska M, Cohen KE, Smith JJ, Lubans DR. Integrating smartphone technology, social support and the outdoor physical environment to improve fitness among adults at risk of, or diagnosed with, type 2 diabetes: findings from the 'eCoFit' randomized controlled trial. Prev Med. 2017;105:404-11.

88. Kovalskys I, Rausch Herscovici C, Indart Rougier P, De Gregorio MJ, Zonis L, Orellana L. Study Protocol of MINI SALTEN: a technology-based multi-component intervention in the school environment targeting healthy habits of first grade children and their parents. BMC Public Health. 2017;17(1):401.

89. Shin DW, Yun JM, Shin JH, Kwon H, Min HY, Joh HK, et al. Enhancing physical activity and reducing obesity through smartcare and financial incentives: a pilot randomized trial. Obesity (Silver Spring). 2017;25(2):302-10.

90. Partridge SR, McGeechan K, Bauman A, Phongsavan P, Allman-Farinelli M. Improved confidence in performing nutrition and physical activity behaviours mediates behavioural change in young adults: mediation results of a randomised controlled mHealth intervention. Appetite. 2017;108:425-33.

91. Armbrust W, Bos G, Wulffraat NM, van Brussel M, Cappon J, Dijkstra PU, et al. Internet program for physical activity and exercise capacity in children with juvenile idiopathic arthritis: a multicenter randomized controlled trial. Arthritis Care Res. 2017;69(7):1040-9.

92. Mehdizadeh A, Nematy M, Khadem-Rezaiyan M, Ghayour-Mobarhan M, Sardar MA, Leis A, et al. A customized intervention program aiming to improve healthy eating and physical activity among preschool children: protocol for a randomized controlled trial (Iran Healthy Start Study). JMIR Res Protoc. 2018;7(12):e11329.

93. Kebede M, Steenbock B, Helmer SM, Sill J, Mollers T, Pischke CR. Identifying evidence-informed physical activity apps: content analysis. JMIR MHealth & UHealth. 2018;6(12):e10314.

94. Simons D, De Bourdeaudhuij I, Clarys P, De Cocker K, Vandelanotte C, Deforche B. Effect and process evaluation of a smartphone app to promote an active lifestyle in lower educated working young adults: cluster randomized controlled trial. JMIR MHealth & UHealth. 2018;6(8):e10003.

95. Herget S, Reichardt S, Grimm A, Petroff D, Kapplinger J, Haase M, et al. High-intensity interval training for overweight adolescents: program acceptance of a media supported intervention and changes in body composition. Int J Environ Res Public Health. 2016;13(11):1099.

96. Hartman SJ, Nelson SH, Cadmus-Bertram LA, Patterson RE, Parker BA, Pierce JP. Technology- and phone-based weight loss intervention: pilot RCT in women at elevated breast cancer risk. Am J Prev Med. 2016;51(5):714-21.

97. Zoellner JM, Hedrick VE, You W, Chen Y, Davy BM, Porter KJ, et al. Effects of a behavioral and health literacy intervention to reduce sugar-sweetened beverages: a randomized-controlled trial. Int J Behav Nutr Phys Act. 2016;13:38.

98. Shin DW, Joh HK, Yun JM, Kwon HT, Lee H, Min H, et al. Design and baseline characteristics of participants in the Enhancing Physical Activity and Reducing Obesity through Smartcare and Financial Incentives (EPAROSFI): a pilot randomized controlled trial. Contemp Clin Trials. 2016;47:115-22.

99. Lonsdale C, Lester A, Owen KB, White RL, Moyes I, Peralta L, et al. An internet-supported physical activity intervention delivered in secondary schools located in low socio-economic status communities: study protocol for the activity and motivation in physical education (AMPED) cluster randomized controlled trial. BMC Public Health. 2016;16:17.

100. Howie EK, Campbell AC, Straker LM. An active video game intervention does not improve physical activity and sedentary time of children at-risk for developmental coordination disorder: a crossover randomized trial. Child Care Health Dev. 2016;42(2):253-60.

101. Biddle SJ, Edwardson CL, Wilmot EG, Yates T, Gorely T, Bodicoat DH, et al. A randomised controlled trial to reduce sedentary time in young adults at risk of type 2 diabetes mellitus: project STAND (Sedentary Time ANd Diabetes). PLoS One. 2015;10(12):e0143398.

102. Sharma SV, Shegog R, Chow J, Finley C, Pomeroy M, Smith C, et al. Effects of the Quest to Lava Mountain computer game on dietary and physical activity behaviors of elementary school children: a pilot group-randomized controlled trial. J Acad Nutr Diet. 2015;115(8):1260-71.

103. Meldrum D, Herdman S, Vance R, Murray D, Malone K, Duffy D, et al. Effectiveness of conventional versus virtual reality-based balance exercises in vestibular rehabilitation for unilateral peripheral vestibular loss: results of a randomized controlled trial. Arch Phys Med Rehabil. 2015;96(7):1319-28.e1.

104. Ptomey LT, Sullivan DK, Lee J, Goetz JR, Gibson C, Donnelly JE. The use of technology for delivering a weight loss program for adolescents with intellectual and developmental disabilities. J Acad Nutr Diet. 2015;115(1):112-8.

105. Fassnacht DB, Ali K, Silva C, Goncalves S, Machado PP. Use of text messaging services to promote health behaviors in children. J Nutr Educ Behav. 2015;47(1):75-80.

106. Cayir Y, Aslan SM, Akturk Z. The effect of pedometer use on physical activity and body weight in obese women. Eur J Sport Sci. 2015;15(4):351-6.

107. Sun H, Gao Y. Impact of an active educational video game on children's motivation, science knowledge, and physical activity. J Sport Health Sci. 2016;5(2):239-45.

108. Allman-Farinelli M, Partridge SR, McGeechan K, Balestracci K, Hebden L, Wong A, et al. A mobile health lifestyle program for prevention of weight gain in young adults (TXT2BFiT): nine-month outcomes of a randomized controlled trial. JMIR MHealth & UHealth. 2016;4(2):e78.

109. Zhang J, Brackbill D, Yang S, Centola D. Efficacy and causal mechanism of an online social media intervention to increase physical activity: results of a randomized controlled trial. Prev Med Rep. 2015;2:651-7.

110. Partridge SR, McGeechan K, Hebden L, Balestracci K, Wong AT, Denney-Wilson E, et al. Effectiveness of a mHealth lifestyle program with telephone support (TXT2BFiT) to prevent unhealthy weight gain in young adults: randomized controlled trial. JMIR MHealth & UHealth. 2015;3(2):e66.

111. Zhou K, He S, Zhou Y, Popeska B, Kuan G, Chen L, et al. Implementation of brain breaks in the classroom and its effects on attitudes towards physical activity in a Chinese school setting. Int J Environ Res Public Health. 2021;18(1):272.

112. Allafi AR. Effects of rewards and pedometer-feedback on children's physical activity: a school-based intervention study. Prog Nutr. 2020;22(1):122-6.

113. Mok MMC, Chin MK, Korcz A, Popeska B, Edginton CR, Uzunoz FS, et al. Brain breaks physical activity solutions in the classroom and on attitudes toward physical activity: a randomized controlled trial among primary students from eight countries. Int J Environ Res Public Health. 2020;17(5):1666.

114. Ulanday KT, Santiago-Torres M, Shi Z, Paul R, Marin-Chollom A, Castellano M, et al. Baseline characteristics of participants enrolled in a randomized controlled trial of a diet and physical activity intervention among Hispanic/Latina breast cancer survivors (in progress). Presented at the 11th AACR Virtual Conference on the Science of Cancer Health Disparities in Racial/Ethnic Minorities and the Medically Underserved; 2-4 October 2020. Cancer Epidemiol Biomark Prev. 2020;29(6 Suppl 1):A034.

115. Salsman J, Tooze JA, Diamond M, Coffman EM, Polzien K, Little-Greene D, et al. A randomized pilot trial of an mhealth physical activity intervention for adolescent and young adult cancer survivors: feasibility, acceptability, and psychological well-being outcomes [Abstract T50]. Presented at 17th Annual Conference of the American Psychosocial Oncology Society; 11-13 March 2020; Portland, OR. Psychooncology. 2020;29(Suppl 1):90-1.

116. Likhitweerawong N, Boonchooduang N, Kittisakmontri K, Chonchaiya W, Louthrenoo O. Short-term outcomes of tablet/smartphone-based (OBEST) application among obese Thai school-aged children and adolescents: a randomized controlled trial. Obesity Medicine. 2020;20:100287.

117. Bovi APD, Di Michele L, Cesari GM, Rimauro I, D'Acunzo I, De Anseris AG, et al. Experience of an obesity and fatty liver disease clinic: application of a personalized mobile based intervention (PediaFit). Presented at the 52nd Annual Meeting of the European Society for Paediatric Gastroenterology Hepatology and Nutrition (ESPGHAN); 5-8 June 2019; Glasgow (Scotland). J Pediatr Gastroenterol Nutr. 2019;68(Suppl 1):1209.

118. Stasinaki A, Brogle B, Buchter D, Shih CHI, Heldt K, White C, et al. A novel digital health intervention improves physical performance in obese youth. Presented at the Joint Annual Meeting Swiss Society of Paediatrics, Swiss Society of Paediatric Surgery and Swiss Society of Child and Adolescent Psychiatry and Psychotherapy; 24-25 May 2018; Lausanne (Switzerland). Swiss Med Wkly. 2018;147(Suppl 228):10S.

119. L'Allemand D, Shih CH, Heldt K, Buchter D, Brogle B, Ruegger D, et al. Design and interim evaluation of a smartphone app for overweight adolescents using a behavioural health intervention platform. Obes Rev. 2018;19(Suppl 1):102.

120. Bos J, Armbrust W, Wulffraat N, Van Brussel M, Cappon J, Dijkstra P, et al. Internet program for physical activity and exercise-capacity in children with juvenile idiopathic arthritis; a multicenter randomized controlled trial. Presented at the 23rd Paediatric Rheumatology European Society Congress; 28 September - 1 October 2016; Genoa (Italy). Pediatric Rheumatology. 2017;15(Suppl 1):67.

121. Livingstone KM, Celis-Morales C, Mathers J. Who benefits most from personalized nutrition? Findings from the pan-European Food4Me randomized controlled trial. Presented at the Annual Meeting at Experimental Biology (EB); 22-26 April 2017; Chicago (IL). FASEB J. 2017;31(1 Suppl 1):963-4.

122. Yoshinaga M, Ogata H, Aoki M, Ito Y, Hamajima T, Miyazaki A, et al. Efficacy of walking as a lifestyle modification approach for childhood obesity. A randomized controlled trial. Presented at the European Society of Cardiology (ESC) Congress; 27-31 August 2016; Rome (Italy). Eur Heart J. 2016;37(Suppl 1):P1402.

123. Markert J, Herget S, Falkenberg C, Blueher S. Feasibility of applying new media in weight maintenance of adolescents with obesity. Presented at the 4th National Obesity Summit; 28 April - 2 May 2015; Toronto (ON) Can J Diabetes. 2015;39(Suppl 1):S34.

124. Lau WC, Zhang S, Maddison R. The effect of a school-based active video game intervention on children's aerobic fitness, physical activity level, and exercise related psychological variables: a preliminary RCT trial. Presented at the 22nd Congress of the European Congress on Obesity (ECO); 6-9 May 2015; Prague (Czech Republic). Obes Facts. 2015;8(Suppl 1):145.

125. Hieftje K, Edelman EJ, Camenga DR, Fiellin LE. Electronic media-based health interventions promoting behavior change in youth: a systematic review. JAMA Pediatr. 2013;167(6):574-80.

126. Cullen KW, Watson K, Baranowski T, Baranowski JH, Zakeri I. Squire's Quest: intervention changes occurred at lunch and snack meals. Appetite. 2005;45(2):148-51.

127. Lubans DR, Morgan PJ, Tudor-Locke C. A systematic review of studies using pedometers to promote physical activity among youth. Prev Med. 2009;48(4):307-15.

128. Yang HJ, Kang JH, Kim OH, Choi M, Oh M, Nam J, et al. Interventions for preventing childhood obesity with smartphones and wearable device: a protocol for a non-randomized controlled trial. Int J Environ Res Public Health. 2017;14(2):184.

129. Franken SCM, Smit CR, Buijzen M. Promoting water consumption on a Caribbean island: an intervention using children's social networks at schools. Int J Environ Res Public Health. 2018;15:713.

130. Teesson M, Newton NC, Slade T, Chapman C, Birrell L, Mewton L, et al. Combined prevention for substance use, depression, and anxiety in adolescence: a cluster-randomised controlled trial of a digital online intervention. Lancet Digit Health. 2020;2(2):e74-84.

131. Neighbors C, DiBello AM, Young CM, Steers MN, Rinker DV, Rodriguez LM, et al. Personalized normative feedback for heavy drinking: an application of deviance regulation theory. Behav Res Ther. 2019;115:73-82.

132. Braitman AL, Henson JM. Personalized boosters for a computerized intervention targeting college drinking: the influence of protective behavioral strategies. J Am Coll Health. 2016;64(7):509-19.

133. Champion KE, Newton NC, Stapinski L, Slade T, Barrett EL, Teesson M. A cross-validation trial of an internet-based prevention program for alcohol and cannabis: preliminary results from a cluster randomised controlled trial. Aust N Z J Psychiatry. 2016;50(1):64-73.

134. Deluca P, Coulton S, Alam MF, Boniface S, Cohen D, Donoghue K, et al. Brief interventions to prevent excessive alcohol use in adolescents at low-risk presenting to emergency departments: three-arm, randomised trial of effectiveness and cost-effectiveness. Int J Drug Policy. 2021;93:103113.

135. Newton NC, Chapman C, Slade T, Birrell L, Healy A, Mather M, et al. A national effectiveness trial of an eHealth program to prevent alcohol and cannabis misuse: responding to the replication crisis. Psychol Med. 2020 June 17 [cited 10.9.21]. Available from: <https://www.cambridge.org/core/journals/psychological-medicine/article/abs/national-effectiveness-trial-of-an-ehealth-program-to-prevent-alcohol-and-cannabis-misuse-responding-to-the-replication-crisis/A8C2EF6A956999A598C1C3DC63589839> [Epub ahead of print].

136. Schwarz EB, Fix M, Lewis C, Muriki M, Satterfield J. Effectiveness of the partywise intervention in improving young women's knowledge and safe consumption of alcohol; a randomized trial. J Gen Intern Med. 2020;35(S101-2).

137. Meyer M, Brudy L, Fuertes-Moure A, Hager A, Oberhoffer-Fritz R, Ewert P, et al. E-health exercise intervention for pediatric patients with congenital heart disease: a randomized controlled trial. J Pediatr. 2021;233:163-8.

138. Cowley ES, Watson PM, Foweather L, Belton S, Mansfield C, Whitcomb-Khan G, et al. Formative evaluation of a home-based physical activity intervention for adolescent girls - the HERizon project: a randomised controlled trial. Children. 2021;8(2):76.

139. Corepal R, Best P, O'Neill R, Kee F, Badham J, Dunne L, et al. A feasibility study of 'The StepSmart Challenge' to promote physical activity in adolescents. Pilot feasibility stud. 2019;5:132.

140. Lee JJ, Nadkarni NV, Teo I, Ozdemir S. The effect of social norm-based intervention with observable behaviour on physical activity among adolescents: a randomized controlled trial. BMC Sports Sci Med Rehabil. 2020;12:52.

141. Sabooteh S, Feizi A, Shekarchizadeh P, Shahnazi H, Mostafavi F. Designing and evaluation of E-health educational intervention on students' physical activity: an application of Pender's health promotion model. BMC Public Health. 2021;21(1):657.

142. Sundgot-Borgen C, Friborg O, Kolle E, Torstveit MK, Sundgot-Borgen J, Engen KME, et al. Does the Healthy Body Image program improve lifestyle habits among high school students? A randomized controlled trial with 12-month follow-up. J Int Med Res. 2020;48(3):300060519889453.

143. Silva KS, Silva JAD, Barbosa Filho VC, Santos PCD, Silveira PMD, Lopes MVV, et al. Protocol paper for the Movimente school-based program: a cluster-randomized controlled trial targeting physical activity and sedentary behavior among Brazilian adolescents. Medicine. 2020;99(31):e21233.

144. Zhamardiy VO, Shkola OM, Okhrimenko IM, Strelchenko OG, Aloshyna AI, Opanasiuk FH, et al. Checking of the methodical system efficiency of fitness technologies application in students' physical education. Wiad Lek. 2020;73(2):332-41.

145. Memon AR, Masood T, Awan WA, Waqas A. The effectiveness of an incentivized physical activity programme (Active Student) among female medical students in Pakistan: a randomized controlled trial. J Pak Med Assoc. 2018;68(10):1438-45.

146. Stephens JD, Yager AM, Allen J. Smartphone technology and text messaging for weight loss in young adults: a randomized controlled trial. J Cardiovasc Nurs. 2017;32(1):39-46.

147. Sharp P, Caperchione C. The effects of a pedometer-based intervention on first-year university students: a randomized control trial. J Am Coll Health. 2016;64(8):630-8.

148. Klausen SH, Andersen LL, Sondergaard L, Jakobsen JC, Zoffmann V, Dideriksen K, et al. Effects of eHealth physical activity encouragement in adolescents with complex congenital heart disease: the PReVaiL randomized clinical trial. Int J Cardiol. 2016;221:1100-6.

149. Anson D, Madras D. Do low step count goals inhibit walking behavior: a randomized controlled study. Clin Rehabil. 2016;30(7):676-85.

150. Jussila AM, Vasankari T, Paronen O, Sievanen H, Tokola K, Vaha-Ypya H, et al. KIDS OUT! Protocol of a brief school-based intervention to promote physical activity and to reduce screen time in a sub-cohort of Finnish eighth graders. BMC Public Health. 2015;15:634.

151. Abraham AA, Chow WC, So HK, Yip BH, Li AM, Kumta SM, et al. Lifestyle intervention using an internet-based curriculum with cell phone reminders for obese Chinese teens: a randomized controlled study. PLoS One. 2015;10(5):e0125673.

152. Dzielska A, Mazur J, Nalecz H, Oblacinska A, Fijalkowska A. Importance of self-efficacy in eating behavior and physical activity change of overweight and non- overweight adolescent girls participating in healthy me: a lifestyle intervention with mobile technology. Nutrients. 2020;12(7):2128.

153. Fijalkowska A, Mazur J, Dzielska A, Nalecz H, Ostrega W, Kleszczewska D, et al. Changes in health-behaviours of 15-year old girls depend on satisfaction with the prophylactic intervention program. Healthy Me Study. Presented at the European Society of Cardiology (ESC) Congress; 31 August - 4 September 2019; Paris (France) Eur Heart J. 2019;40(Suppl 1):P5306.

154. Dabbas M, Lepage G, Boedoz E, Charrat A, Consfroy M, Manh Y. Use of new technologies for the follow-up in adolescent obesity; mobile health intervention (MHI) a randomized controlled trial. Presented at the 9th Europaediatrics Congress of Royal College of Paediatrics and Child Health; 13-15 June 2019; Dublin (Ireland). Arch Dis Child. 2019;104(Suppl 3):A366.

155. Buchter D, Kowatsch T, Tanner A, Vural S, Laimbacher J, Mudespacher A, et al. Treatment of adolescents with under-or overweight by stress regulation exercises: an SMS-supported randomized controlled study*. Presented at European Obesity Summit (EOS): 1st Joint Congress of EASO and IFSO-EC; 1-4 June 2016; Gothenburg Sweden. Obes Facts. 2016;9(Suppl 1):211-2.

156. Morrison BN, DeSouza AM, Voss C, Potts JE, Sandor GG, Harris KC. The use of individualized exercise prescription and activity trackers to promote physical activity in children with congenital heart disease. Presented at the 68th Annual Meeting of the Canadian Cardiovacular Society; 24-27 October 2015; Toronto (ON). Can J Cardiol. 2015;31(10 Suppl 1):S123-4.

157. Da Silva KBB, Ortelan N, Murta SG, Sartori I, Couto RD, Fiaccone RL, et al. Evaluation of the computer-based intervention program stayingfit Brazil to promote healthy eating habits: the results from a school cluster-randomized controlled trial. Int J Environ Res Public Health. 2019;16(10):1674.

158. Laska MN, Lytle LA, Nanney MS, Moe SG, Linde JA, Hannan PJ. Results of a 2-year randomized, controlled obesity prevention trial: effects on diet, activity and sleep behaviors in an at-risk young adult population. Prev Med. 2016;89:230-6.

159. Collins CE, Dewar DL, Schumacher TL, Finn T, Morgan PJ, Lubans DR. 12 month changes in dietary intake of adolescent girls attending schools in low-income communities following the NEAT Girls cluster randomized controlled trial. Appetite. 2014;73:147-55.

160. Kattelmann KK, Bredbenner CB, White AA, Greene GW, Hoerr SL, Kidd T, et al. The effects of Young Adults Eating and Active for Health (YEAH): a theory-based Web-delivered intervention. J Nutr Educ Behav. 2014;46(6):S27-41.

161. Wang J, Baranowski T, Lau PW, Pitkethly AJ, Buday R. Acceptability and applicability of an American health videogame with story for childhood obesity prevention among Hong Kong Chinese children. Games Health J. 2015;4(6):513-9.

162. Rodgers RF, Pernal W, Matsumoto A, Shiyko M, Intille S, Franko DL. Capitalizing on mobile technology to support healthy eating in ethnic minority college students. J Am Coll Health. 2016;64(2):125-32.

163. Folkvord F, de Bruijne M. The effect of the promotion of vegetables by a social influencer on adolescents' subsequent vegetable intake: a pilot study. Int J Environ Res Public Health. 2020;17:2243.

164. Lei S, Scott C, Lerman A, Medina-Inojosa J, Lerman L, de Andrade M, et al. Effectiveness of a weight loss program using digital health in teens and preteens an observational study of 2,825 adolescents. J Am Coll Cardiol. 2020;75(11).

165. Hickman NE, Schaar G. Impact of an educational text message intervention on adolescents' knowledge and high-risk behaviors. Compr Child Adolesc Nurs. 2018;41(1):71-82.

166. Januraga PP, Izwardi D, Crosita Y, Indrayathi PA, Kurniasari E, Sutrisna A, et al. Qualitative evaluation of a social media campaign to improve healthy food habits among urban adolescent females in Indonesia. Public Health Nutr. 2021;24(S2):s98-107.

167. Sundstrom C, Gajecki M, Johansson M, Blankers M, Sinadinovic K, Stenlund-Gens E, et al. Guided and unguided internet-based treatment for problematic alcohol use - a randomized controlled pilot trial. PLoS One. 2016;11(7):e0157817.

168. Hwang J, Lu AS. Narrative and active video game in separate and additive effects of physical activity and cognitive function among young adults. Sci Rep. 2018;8(1):11020.

169. Holzmann SL, Schaefer H, Groh G, Plecher DA, Klinker G, Schauberger G, et al. Effects of the digital game Fit, Food, Fun on nutritional knowledge: a pilot study among German children and adolescents. Proc Nutr Soc. 2020;79(OCE2-2020).

170. Chaput JP, Schwartz C, Boirie Y, Duclos M, Tremblay A, Thivel D. Energy intake adaptations to acute isoenergetic active video games and exercise are similar in obese adolescents. Eur J Clin Nutr. Obs order2015. p. 1267-71.

171. Ruggiero L, Seltzer ED, Dufelmeier D, McGee Montoya A, Chebli P. MyPlate Picks: development and initial evaluation of feasibility, acceptability, and impact of an educational exergame to help promote healthy eating and physical activity in children. Games Health J. 2020;9(3):197-207.

172. Sanatkar S, Heinsch M, Baldwin PA, Rubin M, Geddes J, Hunt S, et al. Factors predicting trial engagement, treatment satisfaction, and health-related quality of life during a web-based treatment and social networking trial for binge drinking and depression in young adults: secondary analysis of a randomized controlled trial. JMIR Mental Health. 2021;8(6):e23986.

173. Shell DF, Newman IM. Effects of a web-based pre-enrollment alcohol brief motivational intervention on college student retention and alcohol-related violations. J Am Coll Health. 2019;67(3):263-74.

174. Gilmore AK, Bountress KE, Selmanoff M, George WH. Reducing heavy episodic drinking, incapacitation, and alcohol-induced blackouts: secondary outcomes of a web-based combined alcohol use and sexual assault risk reduction intervention. Violence Against Women. 2018;24(11):1299-313.

175. Watson J, Toner P, Day E, Back D, Brady LM, Fairhurst C, et al. Youth social behaviour and network therapy (Y-SBNT): adaptation of a family and social network intervention for young people who misuse alcohol and drugs - a randomised controlled feasibility trial. Health Technol Assess. 2017;21(15):1-260.

176. Walsh K, Gilmore AK, Frazier P, Ledray L, Acierno R, Ruggiero KJ, et al. A randomized clinical trial examining the effect of video-based prevention of alcohol and marijuana use among recent sexual assault victims. Alcohol Clin Exp Res. 2017;41(12):2163-72.

177. Bountress KE, Metzger IW, Maples-Keller JL, Gilmore AK. Reducing sexual risk behaviors: secondary analyses from a randomized controlled trial of a brief web-based alcohol intervention for underage, heavy episodic drinking college women. Addict Res Theory. 2017;25(4):302-9.

178. Helmer SM, Muellmann S, Zeeb H, Pischke CR. Development and evaluation of the efficacy of a web-based 'social norms'-intervention for the prevention and reduction of substance use in a cluster-controlled trial conducted at eight German universities. BMC Public Health. 2016;16:252.

179. Bendtsen M, Bendtsen P, Henriksson H, Henriksson P, Mussener U, Thomas K, et al. The mobile health multiple lifestyle behavior interventions across the Lifespan (MOBILE) research program: protocol for development, evaluation, and implementation. JMIR Res Protoc. 2020;9(4):e14894.

180. Thornton LK, Chapman C, Leidl D, Conroy C, Teesson M, Slade T, et al. Climate schools plus: an online, combined student and parent, universal drug prevention program. Internet Interv. 2018;12:36-45.

181. Arman N, Tarakci E, Tarakci D, Kasapcopur O. Effects of video games-based task-oriented activity training (Xbox 360 Kinect) on activity performance and participation in patients with juvenile idiopathic arthritis: a randomized clinical trial. Am J Phys Med Rehabil. 2019;98(3):174-81.

182. Heino MTJ, Knittle K, Haukkala A, Vasankari T, Hankonen N. Simple and rationale-providing SMS reminders to promote accelerometer use: a within-trial randomised trial comparing persuasive messages. BMC Public Health. 2018;18(1):1352.

183. Liu S, Willoughby JF. Do fitness apps need text reminders? An experiment testing goal-setting text message reminders to promote self-monitoring. J Health Commun. 2018;23(4):379-86.

184. Del Corral T, Cebria IIMA, Lopez-de-Uralde-Villanueva I, Martinez-Alejos R, Blanco I, Vilaro J. Effectiveness of a home-based active video game programme in young cystic fibrosis patients. Respiration. 2018;95(2):87-97.

185. Schoeppe S, Alley S, Rebar AL, Hayman M, Bray NA, Van Lippevelde W, et al. Apps to improve diet, physical activity and sedentary behaviour in children and adolescents: a review of quality, features and behaviour change techniques. Int J Behav Nutr Phys Act. 2017;14(1):83.

186. Turner T, Spruijt-Metz D, Wen CK, Hingle MD. Prevention and treatment of pediatric obesity using mobile and wireless technologies: a systematic review. Pediatr Obes. 2015;10(6):403-9.

187. Mackey E, Schweitzer A, Hurtado ME, Hathway J, DiPietro L, Lei KY, et al. The feasibility of an e-mail-delivered intervention to improve nutrition and physical activity behaviors in African American college students. J Am Coll Health. 2015;63(2):109-17.

188. Nguyen B, Shrewsbury VA, O'Connor J, Lau C, Steinbeck KS, Hill AJ, et al. A process evaluation of an adolescent weight management intervention: findings and recommendations. Health Promot Int. 2015;30(2):201-12.

189. Lao LS. Evaluation of a social networking based SNAP-Ed nutrition curriculum on behavior change. MSc [Dissertation]. Kingston (RI): University of Rhode Island; 2011.

190. Lam C, Milne-Ives M, Van Velthoven MH, Meinert E. Internet of things-enabled technologies for weight management in children and adolescents: protocol for a systematic review. JMIR Res Protoc. 2020;9(3):e16930.

191. Teesson M, Champion KE, Newton NC, Kay-Lambkin F, Chapman C, Thornton L, et al. Study protocol of the Health4Life initiative: a cluster randomised controlled trial of an eHealth school-based program targeting multiple lifestyle risk behaviours among young Australians. BMJ OPEN. 2020;10(7):e035662.

192. Champion KE, Newton NC, Spring B, Wafford QE, Parmenter BJ, Teesson M. A systematic review of school-based eHealth interventions targeting alcohol use, smoking, physical inactivity, diet, sedentary behaviour and sleep among adolescents: a review protocol. Syst Rev. 2017;6(1):246.

193. Thomas K, Bendtsen M, Linderoth C, Karlsson N, Bendtsen P, Mussener U. Short message service (SMS)-based intervention targeting alcohol consumption among university students: study protocol of a randomized controlled trial. Trials. 2017;18(1):156.

194. Carreiro S, Chai PR, Carey J, Lai J, Smelson D, Boyer EW. Mhealth for the detection and intervention in adolescent and young adult substance use disorder. Current Addiction Reports. 2018;5(2):110-9.

195. Wright CJ, Dietze PM, Agius PA, Kuntsche E, Room R, Livingston M, et al. An ecological momentary intervention to reduce alcohol consumption in young adults delivered during drinking events: protocol for a pilot randomized controlled trial. JMIR Res Protoc. 2017;6(5):e95.

196. Tebb KP, Erenrich RK, Jasik CB, Berna M, Lester JC, Ozer EM. Use of theory in computer-based interventions to reduce alcohol use among adolescents and young adults. J Adolesc Health. 2016;58(2 Suppl 1):S69-70.

197. Saucedo-Araujo RG, Chillon P, Perez-Lopez IJ, Barranco-Ruiz Y. School-based interventions for promoting physical activity using games and gamification: a systematic review protocol. Int J Environ Res Public Health. 2020;17(14):5186.

198. Lee AM, Chavez S, Bian J, Thompson LA, Gurka MJ, Williamson VG, et al. Efficacy and effectiveness of mobile health technologies for facilitating physical activity in adolescents: scoping review. JMIR MHealth & UHealth. 2019;7(2):e11847.

199. Brannon EE, Cushing CC. Is there an app for that? Translational science of pediatric behavior change for physical activity and dietary interventions: a systematic review. J Pediatr Psychol. 2015;40(4):373-84.

200. Gao Z, Zeng N, McDonough DJ, Su X. A systematic review of active video games on youth's body composition and physical activity. Int J Sports Med. 2020;41(9):561-73.

201. Kim HN, Seo K. Smartphone-based health program for improving physical activity and tackling obesity for young adults: a systematic review and meta-analysis. Int J Environ Res Public Health. 2019;17(1).

202. Ramírez-Granizo IA, Ubago-Jiménez JL, González-Valero G, Puertas-Molero P, San Román-Mata S. The effect of physical activity and the use of active video games: exergames in children and adolescents: a systematic review. Int J Environ Res Public Health. 2020;17(12):4243.

203. Bedendo A, Andrade ALM, Noto AR. [Internet-based alcohol interventions for college students: systematic review]. Rev Panam Salud Publica. 2018;42:e54.

204. Pagkalos I, Kokkinopoulou A, Karampola M, Hassapidou M. Nutri-face: a pilot intervention study on adolescent dietary habits and physical activity levels through Facebook. Acta Paediatr. 2016;106(Suppl 468):23.

205. O'Connor C, Clark A, Clark M, Doherty S, McGregor M, Sadler R, et al. Smartappetite: using innovative technology to promote healthy eating and strengthen the local food economy. Poster presented at the 17th International Congress of Dietetics; 7-10 September 2016; Granada (Spain). Rev Esp Nutricion Humana Diet. 2016;20.

206. Lee CM, Cadigan JM, Kilmer JR, Cronce JM, Suffoletto B, Walter T, et al. Brief Alcohol Screening and Intervention for Community College Students (BASICCS): feasibility and preliminary efficacy of web-conferencing BASICCS and supporting automated text messages. Psychol Addict Behav. 2021 June 10 [cited 10.9.21]. Available from: <https://doi.apa.org/doiLanding?doi=10.1037%2Fadb0000745> [Epub ahead of print].

207. Ahn JS, Lee H, Kim J, Park H, Kim DW, Lee JE. Use of a smartphone application for weightloss versus a paper-based dietary diary: a randomized trial. Presented at the AACR Special Conference on Modernizing Population Sciences in the Digital Age; 19-22 February 2019; San Diego (CA). Cancer Epidemiol Biomark Prev. 2020;29(9 Suppl).

208. Deluca P, Milward J, Drummond C. Randomised controlled trial examining engagement promoting strategies compared to basic strategies within an app targeting harmful drinking in young adults. Presented at the 43rd Annual Poster Abstracts of the Research Society on Alcoholism jointly with the International Society for Biomedical Research on Alcoholism virtual meeting; June 2020. Alcohol Clin Exp Res. 2020;44(Suppl 1):156A.

209. Goncalves MF, Bedendo A, Noto AR. Incentives moderates the effectiveness of a web-based brief intervention among college drinkers. Presented at the 42nd Annual Scientific Meeting of the Research Society on Alcoholism; 22-26 June 2019; Minneapolis (MN). Alcohol Clin Exp Res. 2019;43(Suppl 1):128A.

210. Bernstein MH, Carey KB, Suffoletto B, Stein LAR. Feasibility of a brief text-message intervention. Presented at the 42nd Annual Scientific Meeting of the Research Society on Alcoholism; 22-26 June 2019; Minneapolis (MN). Alcohol Clin Exp Res. 2019;43(Suppl 1):127A.

211. Bedendo A, Andrade ALM, Souza-Formigoni MLO, Noto AR. Motivation moderates the effectiveness of a web-based alcohol brief intervention for college students. Presented at the 42nd Annual Scientific Meeting of the Research Society on Alcoholism; 22-26 June 2019; Minneapolis (MN) Alcohol Clin Exp Res. 2019;43(Suppl 1):127A.

212. Gardner L. Internet-based prevention for alcohol and other drugs: an overview of the universal climate schools prevention programs. J Am Acad Child Adolesc Psychiatry. 2018;57(10 Suppl):S36.

213. Bedendo A, Andrade ALM, Souza-Formigoni MLO, Noto AR. Effectiveness of components of a web-based personalized normative feedback intervention for college drinkers. Presented at 41st Annual Scientific Meeting of the Research Society on Alcoholism; 16-20 June 2018; San Diego (CA). Alcohol Clin Exp Res. 2018;42(Suppl 1):A744.

214. Matteucci A, Reif S, Paschall MJ, DeJong W. Examiningthe combined effects of environmental prevention strategies and alcoholedu for college on college drinking behaviors. Presented at the 41st Annual Scientific Meeting of the Research Society on Alcoholism; 16-20 June 2018; San Diego (CA). Alcohol Clin Exp Res. 2018;42(Suppl 1):253A.

215. Harris SK, Sherritt L, Grubb L, Samuels R, Silva T, Vernacchio L, et al. Practical tools to support adolescent substance abuse prevention in primary care: a multi-site randomized controlled trial of computer-facilitated screening and provider brief advice in the medical office. J Adolesc Health. 2018;62(2 Suppl 1):S13.

216. Chao SH, Glover A, Rufa AK, Boley R, Schueller S, Zalta A, et al. Impact of a smartphone-based intervention on substance use amongst homeless youth. J Am Acad Child Adolesc Psychiatry. 2017;56(10):S276-7.

217. Litt DM, Rhew IC, Fairlie AM, Swanson A, Anderson J, Kaysen D, et al. Evaluating personalized feedback intervention framing with a randomized controlled trial to reduce young adult alcohol-related sexual risk taking. Presented at the 40th Annual Scientific Meeting of the Research Society on Alcoholism; 24-28 June 2017; Denver (CO). Alcohol Clin Exp Res. 2017;41(Suppl 1):73A.

218. Neale ZE, Kidd Donovan K, Hancock L, Aliev F, Savage JE, Cooke ME, et al. Family history as a moderator of the effect of a brief, web-based prevention intervention among college students. Presented at the 40th Annual Scientific Meeting of the Research Society on Alcoholism; 24-28 June 2017; Denver (CO). Alcohol Clin Exp Res. 2017;41(Suppl 1):174A.

219. Bernstein MH, Stein LAR, Neighbors C, Carey KB, Suffoletto B, Wood MD. Efficacy of a 21st birthday text-message intervention with college students. Presented at the 40th annual Scientific Meeting of the Research Society on Alcoholism; 24-28 June; Denver (CO). Alcohol Clin Exp Res. 2017;41(Suppl 1):173A.

220. DiBello AM, Steuer AB, Carey MP, Carey KB. Failure to lower risk of sexual assault after a successful brief alcohol intervention. Presented at the 40th Annual Scientific Meeting of the Research Society on Alcoholism; 24-28 June 2017; Denver (CO). Alcohol Clin Exp Res. 2017;41(Suppl 1):333A.

221. Carey KB, Walsh JL, Merrill JE, Lust SA, Reid AE, Kalichman SC, et al. Effect of email boosters after a brief alcohol intervention for mandated college students. Presented at the 39th Annual Scientific Meeting of the Research Society on Alcoholism; 25-29 June 2016; New Orleans (LA). Alcohol Clin Exp Res. 2016;40(Suppl 1):310A.

222. Cadigan JM, Martens MP, Sher KJ. The efficacy of an event-specific, text-message, personalized drinking feedback intervention. Presented at the 39th Annual Scientific Meeting of the Research Society on Alcoholism; 25-29 June 2016; New Orleans (LA). Alcohol Clin Exp Res. 2016;40(Suppl 1):286A.

223. Earle AM, Boyle SC, LaBrie JW. An app that surreptitiously corrects alcohol norms: the next generation of personalized normative feedback. Presented at the 39th Annual Scientific Meeting of the Research Society on Alcoholism; 25-29 June 2016; New Orleans (LO). Alcohol Clin Exp Res. 2016;40(Suppl 1):111A.

224. Gilmore AK, Bountress KE. Reducing drinking to cope among college women: secondary outcomes of a web-based alcohol use and sexual assault risk reduction intervention. Presented at the 39th Annual Scientific Meeting of the Research Society on Alcoholsm; 25-29 June 2016; New Orleans (LO). Alcohol Clin Exp Res. 2016;40(Suppl 1):70A.

225. Neale ZE, Kidd Donovan K, Hancock L, Aliev F, Salvatore JE, Dick DM. Evaluating a brief web-based prevention intervention for risky alcohol use among college students. Presented at the 39th Annual Scientific Meeting of the Research Society on Alcoholism; 25-29 June 2016; New Orleans (LO). Alcohol Clin Exp Res. 2016;40(Suppl 1):63A.

226. Gelabert Carulla J, Muntaner-Mas A, Palou Sampol P. [Effects of moderate to vigorous intensity interval exercise program on fitness and fatness in 11-and 12-years-old schoolchildren]. Nutr Hosp. 2020;34(3):514-23.

227. Maselli M, Gobbi E, Carraro A. Effectiveness of individual counseling and activity monitors to promote physical activity among university students. J Sports Med Phys Fitness. 2019;59(1):132-40.

228. Ptomey LT, Willis EA, Greene JL, Danon JC, Chumley TK, Washburn RA, et al. The feasibility of group video conferencing for promotion of physical activity in adolescents with intellectual and developmental disabilities. Am J Intellect Dev Disabil. 2017;122(6):525-38.

229. Gao Z, Chen S, Pasco D, Pope Z. A meta-analysis of active video games on health outcomes among children and adolescents. Obes Rev. 2015;16(9):783-94.

230. Quelly SB, Norris AE, DiPietro JL. Impact of mobile apps to combat obesity in children and adolescents: a systematic literature review. J Spec Pediatr Nurs. 2016;21(1):5-17.

231. Santos IKD, Medeiros R, Medeiros JA, Almeida-Neto PF, Sena DCS, Cobucci RN, et al. Active video games for improving mental health and physical fitness - an alternative for children and adolescents during social isolation: an overview. Int J Environ Res Public Health. 2021;18(4):1641.

232. Shin Y, Kim SK, Lee M. Mobile phone interventions to improve adolescents' physical health: a systematic review and meta-analysis. Public Health Nursing (Boston, Mass). 2019;36(6):787-99.

233. Williams WM, Ayres CG. Can active video games improve physical activity in adolescents? A review of RCT. Int J Environ Res Public Health. 2020;17(2):669.

234. Alcantara CM, Silva ANS, Pinheiro P, Queiroz MVO. Digital technologies for promotion of healthy eating habits in teenagers. Rev Bras Enferm. 2019;72(2):513-20.

235. Goodyear VA, Wood G, Skinner B, Thompson JL. The effect of social media interventions on physical activity and dietary behaviours in young people and adults: a systematic review. Int J Behav Nutr Phys Act. 2021;18(1):72.

236. De Cock N, Van Lippevelde W, Vangeel J, Notebaert M, Beullens K, Eggermont S, et al. Feasibility and impact study of a reward-based mobile application to improve adolescents' snacking habits. Public Health Nutr. 2018;21(12):2329-44.

237. Simons M, Brug J, Chinapaw MJM, De Boer M, Seidell J, De Vet E. Replacing non-active video gaming by active video gaming to prevent excessive weight gain in adolescents. PLoS One. 2015;10(7).

238. Lubans DR, Smith JJ, Peralta LR, Plotnikoff RC, Okely AD, Salmon J, et al. A school-based intervention incorporating smartphone technology to improve health-related fitness among adolescents: rationale and study protocol for the NEAT and ATLAS 2.0 cluster randomised controlled trial and dissemination study. BMJ OPEN. 2016;6(6):e010448.

239. Diestelkamp S, Schulz AL, Thomasius R. [Technology-based interventions for alcohol prevention among children and adolescents]. Bundesgesundheitsblatt Gesundheitsforschung Gesundheitsschutz. 2021;64(6):714-21.

240. Bedendo A, McCambridge J, Gaume J, Souza AAL, Formigoni M, Noto AR. Components evaluation of a web-based personalized normative feedback intervention for alcohol use among college students: a pragmatic randomized controlled trial with a dismantling design. Addiction. 2020;115(6):1063-74.

241. Martinez-Montilla JM, Mercken L, de Vries H, Candel M, Lima-Rodriguez JS, Lima-Serrano M. A web-based, computer-tailored intervention to reduce alcohol consumption and binge drinking among Spanish adolescents: cluster randomized controlled trial. J Med Internet Res. 2020;22(1):e15438.

242. Bedendo A, Ferri CP, de Souza AAL, Andrade ALM, Noto AR. Pragmatic randomized controlled trial of a web-based intervention for alcohol use among Brazilian college students: motivation as a moderating effect. Drug Alcohol Depend. 2019;199:92-100.

243. Zamboanga BL, Merrill JE, Olthuis JV, Milroy JJ, Sokolovsky AW, Wyrick DL. Secondary effects of myPlaybook on college athletes' avoidance of drinking games or pregaming as a protective behavior strategy: a multisite randomized controlled study. Soc Sci Med. 2019;228:135-41.

244. Braitman AL, Lau-Barraco C. Personalized boosters after a computerized intervention targeting college drinking: a randomized controlled trial. Alcohol Clin Exp Res. 2018;42(9):1735-47.

245. Bernstein MH, Stein LAR, Neighbors C, Suffoletto B, Carey KB, Ferszt G, et al. A text message intervention to reduce 21st birthday alcohol consumption: evaluation of a two-group randomized controlled trial. Psychol Addict Behav. 2018;32(2):149-61.

246. Gilbertson RJ, Norton TR, Beery SH, Lee KR. Web-based alcohol intervention in first-year college students: efficacy of full-program administration prior to second semester. Subst Use Misuse. 2018;53(6):1021-9.

247. Davies EL, Lonsdale AJ, Hennelly SE, Winstock AR, Foxcroft DR. Personalized digital interventions showed no impact on risky drinking in young adults: a pilot randomized controlled trial. Alcohol Alcohol. 2017;52(6):671-6.

248. Leeman RF, DeMartini KS, Gueorguieva R, Nogueira C, Corbin WR, Neighbors C, et al. Randomized controlled trial of a very brief, multicomponent web-based alcohol intervention for undergraduates with a focus on protective behavioral strategies. J Consult Clin Psychol. 2016;84(11):1008-15.

249. Linowski SA, DiFulvio GT, Fedorchak D, Puleo E. Effectiveness of an electronic booster session delivered to mandated students. Int Q Community Health Educ. 2016;36(2):123-9.

250. Jander A, Crutzen R, Mercken L, Candel M, de Vries H. Effects of a web-based computer-tailored game to reduce binge drinking among Dutch adolescents: a cluster randomized controlled trial. J Med Internet Res. 2016;18(2):e29.

251. Caudwell KM, Mullan BA, Hagger MS. Combining motivational and volitional approaches to reducing excessive alcohol consumption in pre-drinkers: a theory-based intervention protocol. BMC Public Health. 2016;16:45.

252. Strohman AS, Braje SE, Alhassoon OM, Shuttleworth S, Van Slyke J, Gandy S. Randomized controlled trial of computerized alcohol intervention for college students: role of class level. Am J Drug Alcohol Abuse. 2016;42(1):15-24.

253. Berman AH, Gajecki M, Sinadinovic K, Andersson C. Mobile interventions targeting risky drinking among university students: a review. Current Addiction Reports. 2016;3:166-74.

254. Valeriani F, Protano C, Marotta D, Liguori G, Romano Spica V, Valerio G, et al. Exergames in childhood obesity treatment: a systematic review. Int J Environ Res Public Health. 2021;18(9):4938.

255. Lee JE, Zeng N, Oh Y, Lee D, Gao Z. Effects of Pokemon GO on physical activity and psychological and social outcomes: a systematic review. J Clin Med. 2021;10(9).

256. Brannon EE, Cushing CC, Walters RW, Crick C, Noser AE, Mullins LL. Goal feedback from whom? A physical activity intervention using an N-of-1 RCT. Psychol Health. 2018;33(6):701-12.

257. Muller AM, Khoo S. Interdisciplinary, child-centred collaboration could increase the success of potentially successful Internet-based physical activity interventions. Acta Paediatr. 2016;105(3):234-43.

258. Garde A, Umedaly A, Abulnaga SM, Robertson L, Junker A, Chanoine JP, et al. Assessment of a mobile game ("MobileKids Monster Manor") to promote physical activity among children. Games Health J. 2015;4(2):149-58.

259. Creaser AV, Clemes SA, Costa S, Hall J, Ridgers ND, Barber SE, et al. The acceptability, feasibility and effectiveness of wearable activity trackers for increasing physical activity in children and adolescents: a systematic review. Int J Environ Res Public Health. 2021;18(12):6211.

260. Chung AE, Skinner AC, Hasty SE, Perrin EM. Tweeting to health: a novel mhealth intervention using fitbits and twitter to foster healthy lifestyles. Clin Pediatr (Phila). 2017;56(1):26-32.

261. Simons M, Chinapaw MJ, Brug J, Seidell J, de Vet E. Associations between active video gaming and other energy-balance related behaviours in adolescents: a 24-hour recall diary study. Int J Behav Nutr Phys Act. 2015;12:32.

262. Militello LK, Kelly SA, Melnyk BM. Systematic review of text-messaging interventions to promote healthy behaviors in pediatric and adolescent populations: implications for clinical practice and research. Worldviews Evid Based Nurs. 2012;9(2):66-77.

263. Thompson D, Baranowski T, Baranowski J, Cullen K, Jago R, Watson K, et al. Boy Scout 5-a-Day Badge: outcome results of a troop and Internet intervention. Prev Med. 2009;49(6):518-26.

264. Ezendam NP, Brug J, Oenema A. Evaluation of the Web-based computer-tailored FATaintPHAT intervention to promote energy balance among adolescents: results from a school cluster randomized trial. Arch Pediatr Adolesc Med. 2012;166(3):248-55.

265. Smith JJ, Morgan PJ, Plotnikoff RC, Dally KA, Salmon J, Okely AD, et al. Smart-phone obesity prevention trial for adolescent boys in low-income communities: the ATLAS RCT. Pediatrics. 2014;134(3):e723-31.

266. Baranowski T, Baranowski J, Cullen KW, Marsh T, Islam N, Zakeri I, et al. Squire's Quest! Dietary outcome evaluation of a multimedia game. Am J Prev Med. 2003;24(1):52-61.

267. Whittemore R, Jeon S, Grey M. An internet obesity prevention program for adolescents. J Adolesc Health. 2013;52(4):439-47.

268. Magalhaes P, Silva C, Pereira B, Figueiredo G, Guimaraes A, Pereira A, et al. An online-based intervention to promote healthy eating through self-regulation among children: study protocol for a randomized controlled trial. Trials. 2020;21(1):786.

269. Chagas C, Pontes ESTB, Reffatti LM, Botelho RBA, Toral N. Rango Cards, a digital game designed to promote a healthy diet: a randomized study protocol. BMC Public Health. 2018;18(1):910.

270. Belogianni K, Ooms A, Ahmed H, Nikoletou D, Grant R, Makris D, et al. Rationale and design of an online educational program using game-based learning to improve nutrition and physical activity outcomes among university students in the United Kingdom. J Am Coll Nutr. 2019;38(1):23-30.

271. Melo GR, Correa Lima S, C MDSC, Nakano EY, Toral N. Tailored smartphone intervention to promote healthy eating among Brazilian adolescents: a randomised controlled trial protocol. BMJ OPEN. 2020;10(10):e038896.

272. Bonar EE, Schneeberger DM, Bourque C, Bauermeister JA, Young SD, Blow FC, et al. Social media interventions for risky drinking among adolescents and emerging adults: protocol for a randomized controlled trial. JMIR Res Protoc. 2020;9(5):e16688.

273. Haug S, Castro RP, Wenger A, Schaub MP. Efficacy of a smartphone-based coaching program for addiction prevention among apprentices: study protocol of a cluster-randomised controlled trial. BMC Public Health. 2020;20(1):1910.

274. Pedersen ER, D'Amico EJ, LaBrie JW, Farris C, Klein DJ, Griffin BA. An online alcohol and risky sex prevention program for college students studying abroad: study protocol for a randomized controlled trial. Addict Sci Clin Pract. 2019;14(1):32.

275. Diestelkamp S, Wartberg L, Kaess M, Bauer S, Rummel-Kluge C, Becker K, et al. Effectiveness of a web-based screening and brief intervention with weekly text-message-initiated individualised prompts for reducing risky alcohol use among teenagers: study protocol of a randomised controlled trial within the ProHEAD consortium. Trials. 2019;20(1):73.

276. Haug S, Paz Castro R, Wenger A, Schaub MP. Efficacy of a mobile phone-based life-skills training program for substance use prevention among adolescents: study protocol of a cluster-randomised controlled trial. BMC Public Health. 2018;18(1):1102.

277. Lima-Serrano M, Martinez-Montilla JM, Lima-Rodriguez JS, Mercken L, de Vries H. Design, implementation and evaluation of a web-based computer-tailored intervention to prevent binge drinking in adolescents: study protocol. BMC Public Health. 2018;18(1):449.

278. Ramo DE, Kaur M, Corpuz ES, Satre DD, Delucchi K, Brown SA, et al. Using Facebook to address smoking and heavy drinking in young adults: protocol for a randomized, controlled trial. Contemp Clin Trials. 2018;68:52-60.

279. Stapinski LA, Prior K, Newton NC, Deady M, Kelly E, Lees B, et al. Protocol for the Inroads Study: a randomized controlled trial of an internet-delivered, cognitive behavioral therapy-based early intervention to reduce anxiety and hazardous alcohol use among young people. JMIR Res Protoc. 2019;8(4):e12370.

280. Riordan BC, Moradi S, Carey KB, Conner TS, Jang K, Reid KE, et al. Effectiveness of a combined web-based and ecological momentary intervention for incoming first-year university students: protocol for a 3-arm randomized controlled trial. JMIR Res Protoc. 2018;7(5):e10164.

281. Pedersen ER, Marshall GN, Schell TL. Study protocol for a web-based personalized normative feedback alcohol intervention for young adult veterans. Addict Sci Clin Pract. 2016;11(1):6.

282. Ip P, Chan KL, Chow CB, Lam TH, Ho SY, Wong WH, et al. An internet-based intervention to promote alcohol-related attitudinal and behavioral change among adolescents: protocol of a cluster randomized controlled trial. JMIR Res Protoc. 2016;5(2):e103.

283. Debenham J, Birrell L, Champion K, Newton N. Study protocol for a cluster randomised controlled trial of The Illicit Project, a digital, neuroscience-based substance use intervention for secondary school students. Contemp Clin Trials. 2021;107:106467.

284. Trottier CF, Lieffers JRL, Johnson ST, Mota JF, Gill RK, Prado CM. The impact of a web-based mindfulness, nutrition, and physical activity platform on the health status of first-year university students: protocol for a randomized controlled trial. JMIR Res Protoc. 2021;10(3):e24534.

285. Oppezzo M, Tremmel J, Desai M, Baiocchi M, Ramo D, Cullen M, et al. Twitter-based social support added to Fitbit self-monitoring for decreasing sedentary behavior: protocol for a randomized controlled pilot trial with female patients from a women's heart clinic. JMIR Res Protoc. 2020;9(12):e20926.

286. Fisher D, Louw Q, Thabane L. Sedentariness and back health in Western Cape primary school students: protocol for a pragmatic stepped-wedge feasibility randomized controlled trial. JMIR Res Protoc. 2020;9(11):e18522.

287. Michalsen H, Wangberg SC, Hartvigsen G, Jaccheri L, Muzny M, Henriksen A, et al. Physical activity with tailored mhealth support for individuals with intellectual disabilities: protocol for a randomized controlled trial. JMIR Res Protoc. 2020;9(6):e19213.

288. Moore JB, Dilley JR, Singletary CR, Skelton JA, Miller DP, Jr., Heboyan V, et al. A clinical trial to increase self-monitoring of physical activity and eating behaviors among adolescents: protocol for the ImPACT feasibility study. JMIR Res Protoc. 2020;9(6):e18098.

289. Partridge SR, Raeside R, Singleton AC, Hyun K, Latham Z, Grunseit A, et al. Text message behavioral intervention for teens on eating, physical activity and social wellbeing (TEXTBITES): protocol for a randomized controlled trial. JMIR Res Protoc. 2020;9(2):e16481.

290. Szabo-Reed AN, Washburn RA, Greene JL, Ptomey LT, Gorczyca A, Lee RH, et al. Physical activity across the curriculum (PAAC3): testing the application of technology delivered classroom physical activity breaks. Contemp Clin Trials. 2020;90:105952.

291. Wang X, Hunter DJ, Robbins S, Capistrano S, Duong V, Melo L, et al. Participatory health through behavioural engagement and disruptive digital technology for postoperative rehabilitation: protocol of the PATHway trial. BMJ OPEN. 2021;11(1):e041328.

292. Masse LC, Vlaar J, Macdonald J, Bradbury J, Warshawski T, Buckler EJ, et al. Aim2Be mHealth intervention for children with overweight and obesity: study protocol for a randomized controlled trial. Trials. 2020;21(1):132.

293. Puigdomenech E, Martin A, Lang A, Adorni F, Gomez SF, McKinstry B, et al. Promoting healthy teenage behaviour across three European countries through the use of a novel smartphone technology platform, PEGASO fit for future: study protocol of a quasi-experimental, controlled, multi-Centre trial. BMC Med Inform Decis Mak. 2019;19(1):278.

294. Ptomey LT, Washburn RA, Lee J, Greene JL, Szabo-Reed AN, Sherman JR, et al. Individual and family-based approaches to increase physical activity in adolescents with intellectual and developmental disabilities: rationale and design for an 18 month randomized trial. Contemp Clin Trials. 2019;84:105817.

295. Liang W, Duan YP, Shang BR, Wang YP, Hu C, Lippke S. A web-based lifestyle intervention program for Chinese college students: study protocol and baseline characteristics of a randomized placebo-controlled trial. BMC Public Health. 2019;19(1):1097.

296. Podina IR, Fodor LA, Cosmoiu A, Boian R. An evidence-based gamified mHealth intervention for overweight young adults with maladaptive eating habits: study protocol for a randomized controlled trial. Trials. 2017;18(1):592.

297. Ridgers ND, Timperio A, Brown H, Ball K, Macfarlane S, Lai SK, et al. A cluster-randomised controlled trial to promote physical activity in adolescents: the Raising Awareness of Physical Activity (RAW-PA) study. BMC Public Health. 2017;17(1):6.

298. Jones Bell M, Zeiler M, Herrero R, Kuso S, Nitsch M, Etchemendy E, et al. Healthy Teens @ School: evaluating and disseminating transdiagnostic preventive interventions for eating disorders and obesity for adolescents in school settings. Internet Interv. 2019;16:65-75.

299. Donnelly JE, Ptomey LT, Goetz JR, Sullivan DK, Gibson CA, Greene JL, et al. Weight management for adolescents with intellectual and developmental disabilities: rationale and design for an 18 month randomized trial. Contemp Clin Trials. 2016;51:88-95.

300. Lonsdale C, Sanders T, Cohen KE, Parker P, Noetel M, Hartwig T, et al. Scaling-up an efficacious school-based physical activity intervention: Study protocol for the 'Internet-based Professional Learning to help teachers support Activity in Youth' (iPLAY) cluster randomized controlled trial and scale-up implementation evaluation. BMC Public Health. 2016;16(1):873.

301. Tzelepis F, Paul CL, Wiggers J, Kypri K, Bonevski B, McElduff P, et al. Targeting multiple health risk behaviours among vocational education students using electronic feedback and online and telephone support: protocol for a cluster randomised trial. BMC Public Health. 2015;15:550.

302. Hagger MS, Wong GG, Davey SR. A theory-based behavior-change intervention to reduce alcohol consumption in undergraduate students: trial protocol. BMC Public Health. 2015;15:306.

303. Hurley JC, Hollingshead KE, Todd M, Jarrett CL, Tucker WJ, Angadi SS, et al. The Walking Interventions Through Texting (WalkIT) Trial: rationale, design, and protocol for a factorial randomized controlled trial of adaptive interventions for overweight and obese, inactive adults. JMIR Res Protoc. 2015;4(3):e108.

304. Downs J, Lotan M, Elefant C, Leonard H, Wong K, Buckley N, et al. Implementing telehealth support to increase physical activity in girls and women with Rett syndrome - ActivRett: protocol for a waitlist randomised controlled trial. BMJ OPEN. 2020;10(12):e042446.

305. Middelweerd A, Te Velde SJ, Klein MCA, Van Halteren AT, Brug J. Active2Gether: innovative and smart coaching strategies to promote physical activity. A research protocol. Presented at the 8th European Congress of Epidemiology; 25-27 June 2015; Maastricht (the Netherlands). Eur J Epidemiol. 2015;30(8):874-5.
