# Supplementary material for: Digital interventions to moderate alcohol consumption in young people: a Cancer Prevention Europe overview of systematic reviews
Source: Front Digit Health. 2023 May 23;5:1178407. doi: 10.3389/fdgth.2023.1178407 (PMC10243367; doi:10.3389/fdgth.2023.1178407)
Supplement: Supplementary file 4 [file Datasheet4.docx]

**Supplementary file 4. List of abbreviations**

AUDIT Alcohol Use Disorders Identification Test

CDI computer-delivered intervention

CDSR Cochrane Database of Systematic Reviews

CI confidence interval

CPE Cancer Prevention Europe

DARE Database of Abstracts of Reviews of Effects

e-SBI electronic screening and brief intervention

HTA Health Technology Assessment

GRADE Grading of Recommendations, Assessment, Development and Evaluations

IARC International Agency for Research on Cancer

mHealth mobile health

MD mean difference

OR odds ratio

PICO population, intervention, comparator, and outcomes

PRISMA Preferred Reporting Items for Systematic Reviews and Meta-Analyses

RCT randomised controlled trial

RoB risk of bias

ROBIS the Risk of Bias Assessment Tool for Systematic Reviews

SMD standardised mean difference

SMS short messaging service

UK the United Kingdom

US the United States of America

VR virtual reality

WCRF World Cancer Research Fund

WHO World Health Organization
